# Partial‐Single‐Atom, Partial‐Nanoparticle Composites Enhance Water Dissociation for Hydrogen Evolution

**DOI:** 10.1002/advs.202001881

**Published:** 2020-11-25

**Authors:** Chun Hu, Erhong Song, Maoyu Wang, Wei Chen, Fuqiang Huang, Zhenxing Feng, Jianjun Liu, Jiacheng Wang

**Affiliations:** ^1^ State Key Laboratory of High Performance Ceramics and Superfine Microstructure Shanghai Institute of Ceramics Chinese Academy of Sciences Shanghai 200050 China; ^2^ Center of Materials Science and Optoelectronics Engineering University of Chinese Academy of Sciences Beijing 100049 China; ^3^ Center of Materials Science and Optoelectronics Engineering University of the Chinese Academy of Sciences Beijing 100049 China; ^4^ School of Chemical Biological, and Environmental Engineering Oregon State University Corvallis OR 97331 USA; ^5^ Department of Mechanical Materials and Aerospace Engineering Illinois Institute of Technology Chicago IL 60616 USA

**Keywords:** electrocatalysis, multiple sites, single‐atom catalysts, theoretical calculations, water dissociation

## Abstract

The development of an efficient electrocatalyst toward the hydrogen evolution reaction (HER) is of significant importance in transforming renewable electricity to pure and clean hydrogen by water splitting. However, the construction of an active electrocatalyst with multiple sites that can promote the dissociation of water molecules still remains a great challenge. Herein, a partial‐single‐atom, partial‐nanoparticle composite consisting of nanosized ruthenium (Ru) nanoparticles (NPs) and individual Ru atoms as an energy‐efficient HER catalyst in alkaline medium is reported. The formation of this unique composite mainly results from the dispersion of Ru NPs to small‐size NPs and single atoms (SAs) on the Fe/N codoped carbon (Fe–N–C) substrate due to the thermodynamic stability. The optimal catalyst exhibits an outstanding HER activity with an ultralow overpotential (9 mV) at 10 mA cm^−2^ (*η*_10_), a high turnover frequency (8.9 H_2_ s^−1^ at 50 mV overpotential), and nearly 100% Faraday efficiency, outperforming the state‐of‐the‐art commercial Pt/C and other reported HER electrocatalysts in alkaline condition. Both experimental and theoretical calculations reveal that the coexistence of Ru NPs and SAs can improve the hydride coupling and water dissociation kinetics, thus synergistically enhancing alkaline hydrogen evolution performance.

## Graphical abstract

A nanocomposite of partial‐single‐atom and partial‐nanoparticle formed within the Fe–N–C matrix serves as a multiple‐site electrocatalyst toward hydrogen evolution reaction with an ultralow overpotential of 9 mV to achieve 10 mA cm^−2^, a high turnover frequency, and ≈100% Faradaic efficiency. Theoretical calculations reveal that ruthenium single‐atoms effectively facilitate water dissociation, and ruthenium nanoparticles promote hydrogen desorption.

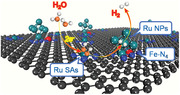

Hydrogen has been regarded as an alternative to fossil fuel due to its clean and sustainable merits. Among the numerous approaches available, water electrolysis could transform the electricity from the intermittent solar and wind power to produce hydrogen.^[^ ^1^, ^2^, ^3^ ^]^ The electrochemical hydrogen evolution reaction (HER) at the cathode is a fundamental process in water splitting. Till now, platinum (Pt) is usually recognized as the most efficient HER electrocatalyst in acid medium owing to its moderate hydrogen binding energy.^[^ ^4^ ^]^ However, the high cost and scarcity of Pt hinder its large‐scale applications.^[^ ^5^ ^]^

On the other hand, alkaline liquid electrolyzer technology has been commercially used because of the overall low cost of various components.^[^ ^6^ ^]^ Whereas, the activity of Pt in alkaline condition is about two to three orders of magnitude lower than that in acid.^[^ ^7^ ^]^ Previous studies have clearly expounded the significant steps in hydrogen evolution in alkaline media. The fist‐step water dissociation (H_2_O + e^−^ → H* + OH^−^, where H* represents adsorbed H on active site *) is followed by either Tafel step (2H* → H_2_) or Heyrovsky step (H_2_O + H* + e^−^ → H_2_ + OH^−^).^[^ ^8^, ^9^ ^]^ Thus, the development of highly efficient and stable electrocatalysts that have a low water dissociation barrier as well as appropriate hydrogen adsorption/desorption strength is highly essential in industrial applications.

In this regard, non‐Pt noble metals such as ruthenium (Ru), palladium (Pd), rhodium (Rh), have been increasingly studied due to their considerable performance for alkaline HER.^[^ ^4^, ^10^, ^11^, ^12^ ^]^ Among these metals, Ru has a comparable HER activity to Pt in alkaline medium, which has been reported in the forms of single atoms (SAs), nanoparticles (NPs), alloys, and oxides.^[^ ^4^, ^11^, ^13^, ^14^, ^15^, ^16^ ^]^ Some researches attributed the high HER activity to Ru NPs.^[^ ^4^, ^17^, ^18^, ^19^ ^]^ For example, Baek et al. found the Ru NPs in the holes of nitrogenated carbon (Ru@C_2_N) can speed the dissociation of water, which could provide more intermediate protons.^[^ ^4^ ^]^ Whereas, some studies indicated the remarkable performance of Ru is derived from the SAs rather than NPs.^[^ ^11^, ^20^, ^21^ ^]^ For instance, Chen et al. found the Ru SAs coordinated with N and C (RuC*_x_*N*_y_*) are more beneficial to water dissociation than Ru NPs because of the lower kinetic barrier.^[^ ^11^ ^]^ This inspires the idea of investigating the exact roles of Ru NPs and SAs in alkaline HER.

Herein, we designed a partial‐single‐atom, partial‐nanoparticle nanocomposite via the coupling of Ru SAs and Ru NPs on the Fe/N codoped carbon (Fe–N–C) substrate, and further evaluated the influence of both SAs and NPs on the HER performance. The existence of Fe SAs coordinated with N groups (Fe–N) in the carbon matrix could disperse the large‐sized Ru NPs into Ru SAs stabilized by N groups (Ru–N_4_) and smaller Ru NPs. Moreover, the Fe–N groups could effectively adjust the electronic distribution of these Ru NPs, thus achieving the optimal Δ*G*_H*_. Density functional theory (DFT) simulation reveals that, the Ru–N_4_ SA moieties facilitate the splitting of water molecules and the generation of hydrogen adsorbates that then recombine into hydrogen molecules on the nearby smaller Ru NPs. Both of the Ru SAs with a low water dissociation barrier and Ru NPs with a proper hydrogen adsorption/desorption strength synergistically enhance hydrogen evolution performance in alkaline condition.

*Synthesis and Structural Identification*: Generally, the size and placement of metal NPs can generate distinct catalytic activity.^[^ ^11^, ^20^, ^22^ ^]^ In this work, we prepared the Ru/C, Ru/N–C and Ru/Fe–N–C nanocomposites via a two‐step process (see details in the Experimental Section), as shown in **Figure** **1**a. Abundant voids originating from the removal of colloidal silica were observed in Ru/Fe–N–C (Figure S2, Supporting Information). The resulting Ru/Fe–N–C consists of C, N, Fe, and Ru elements, as well as a small amount of O element derived from trapped moisture and/or edged oxygen‐containing groups (Figure S3 and Table S1, Supporting Information). Ultrafine Ru NPs are dispersed within porous Fe–N–C matrix (Figure S4, Supporting Information). The lattice spacing of Ru NPs is 0.232 and 0.212 nm, which is attributed to the (100) and (002) planes of Ru, respectively (Figure 1b). There are many Ru SAs around Ru NPs (Figure 1c and Figure S5, Supporting Information), and this has also been observed in the previous research.^[^ ^11^, ^20^ ^]^ The Ru content of Ru/Fe–N–C is 4.92 wt%, determined by the inductively coupled plasma (ICP) analysis. The N_2_ adsorption/desorption measurements confirm a narrow mesopore size distribution, a large surface area of 810 m^2^ g^−1^ and pore volume of 1.8 cm^3^ g^−1^ for Ru/Fe–N–C (Figure S6 and Table S2, Supporting Information). With the increasing of Ru content, the particle size of Ru becomes larger (Figure S7, Supporting Information). The high‐angle annular dark‐field scanning transmission electron microscopy (HAADF‐STEM) image and the corresponding energy dispersive X‐ray (EDX) mapping were employed to analyze the distribution of Ru, Fe, N, and C elements. The Fe component is either abundant (zone I) or deficient (zone II) around Ru NPs (Figure 1d), and N moiety is homogeneously distributed within the carbon matrix. Furthermore, no aggregated Fe particles are observed (Figure 1d), so as to Ru‐free Fe–N–C sample (Figures S8–S10, Supporting Information).

**Figure 1:**
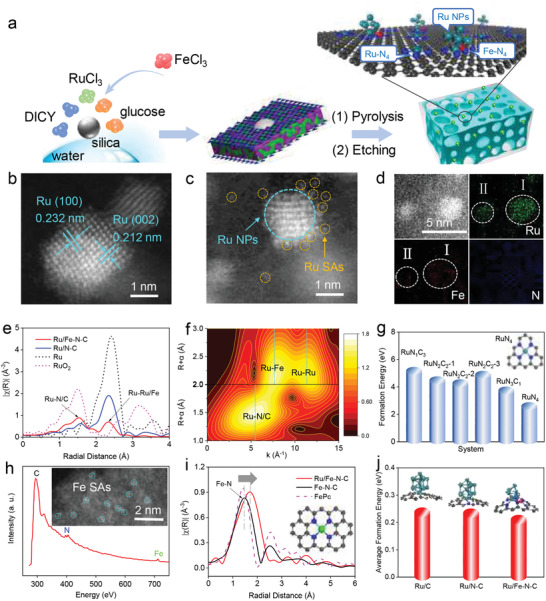
Morphology and structure of Ru/Fe–N–C. a) Schematic procedures for the synthesis of Ru/Fe–N–C. b) High‐angle annular dark‐field scanning TEM (HAADF‐STEM) image of Ru NPs. c) HAADF‐STEM image implies the presence of Ru NPs and Ru SAs in Ru/Fe–N–C (dashed aqua green circle indicates Ru NPs, while orange ones show the Ru SAs). d) HAADF‐STEM image and the corresponding elemental mapping of Ru, Fe, and N. e) Fourier transformed EXAFS *k* ^3^‐weighted *χ*(*R*) function spectra of Ru in Ru/Fe–N–C, Ru/N–C, Ru, and RuO_2_. f) Wavelet transforms for the *k* ^2^‐weighted Ru K‐edge EXAFS in Ru/Fe–N–C. g) The calculated formation energy of RuN*_x_*C*_y_* (*x* +*y* ≤ 4) structures in Ru/Fe–N–C. h) Electron energy loss spectroscopic spectra (EELS) and corresponding HAADF‐STEM image (inset) of Fe–N–C. i) Fourier transformed EXAFS *k* ^3^‐weighted *χ*(*R*) function spectra of Fe in Fe–N–C, Ru/Fe–N–C, and FePc. j) The calculated average formation energy of three predicted structures (Ru/C, Ru/N–C, and Ru/Fe–N–C).

To further understanding of the Ru structure, Ru K‐edge X‐ray absorption spectroscopy (XAS) was performed (Figure S12, Supporting Information).^[^ ^23^, ^24^ ^]^ As shown in Figure 1e, the Fourier‐transform Ru K‐edge extended X‐ray absorption fine structure (EXAFS) of Ru/Fe–N–C exhibits two main peaks. The peak of ≈1.5 Å is Ru–N/C scattering due to the existing of Ru SAs,^[^ ^16^ ^]^ and the other at ≈2.4 Å is associated with Ru–Ru scattering caused by the formation of Ru NPs. Meanwhile, Ru/Fe–N–C has a smaller size of Ru NPs than Ru/N–C, as reflected by the lower Ru–Ru scattering intensity.^[^ ^25^, ^26^ ^]^ Specifically, model‐based EXAFS fitting further reveals that the ratio of Ru–Ru between Ru/N–C and Ru/Fe–N–C is ≈2.17 (Figure S13–S14 and Table S3, Supporting Information), which confirms the larger cluster size in Ru/N–C. From the EXAFS fitting result for Ru/Fe–N–C, we notice that the Ru–N coordination is oversaturated (higher than 4 in coordination number) even considering the EXAFS fitting error if we do not include any Ru–Fe bond (Ru–Fe interaction), as shown in Figure S13 and Table S3 (Supporting Information). We believe that the Ru NPs could be not simply dispersed on the Fe–N–C surface, and it should interact with Fe–N species. The wavelet transfer of Ru K‐edge EXAFS confirms that there is some other Ru‐scattering around 2.4 Å instead of only Ru–Ru scattering which could be Ru–Fe (Figure 1f). When including the Ru–Fe bonds in our model, the fitting quality for Ru/Fe–N–C EXAFS is much improved (Figure S13 and Table S3, Supporting Information).

To identify the most possible atom‐dispersion structure, a series of RuN*_x_*C*_y_* configurations (*x* + *y* ≤ 4) including 2, 3, or 4 coordinates were calculated by using DFT method. Their structural stability is determined by comparing their formation energies of single Ru insertion into different defected configurations (Figure 1g; Figure S19 and Table S4, Supporting Information). The formation energies of RuN*_x_*C*_y_* (*x* + *y* ≤ 4) are in agreement with the previously reported results.^[^ ^11^, ^21^, ^27^, ^28^ ^]^ The calculation results also explain the experimental observation of Ru–N and Ru–C bonding.

On the other hand, to eliminate the influence of Ru element, HAADF‐STEM and electron energy loss spectroscopic (EELS) measurements were conducted in pure Fe–N–C samples. As shown in Figure 1h, Fe exists in the formation of single atomic configurations. Quantitative analysis of Fourier transformed Fe K‐edge EXAFS further reveals the presence of Fe SA sites in Fe–N–C. Since it is hard for EXAFS to distinguish the elements that are close in atomic number, we start with DFT‐suggested best models to refine the EXAFS results, and in turn these fitted parameters will feed back to DFT to further confirm the local configurations. In addition, the previous literatures^[^ ^29^, ^30^, ^31^, ^32^ ^]^ suggest that Fe prefers to form SA sites anchored on N for high activity. Finally, the model‐based fitting of Fe with standard FeN_4_ structure reproduce our EXAFS spectrum perfectly, which gives a mean bond length of 2.03 ± 0.02 Å for Fe–N. The Fe–N bond length in Ru/Fe–N–C is evidently longer than that in Fe–N–C, which may originate from strong interaction of Ru NPs and Fe–N group (Figure 1i). Based on the interaction of Ru NPs and Fe–N from our above discussions, the second scattering peak in wavelet transfer Ru EXAFS is assigned to Ru–Fe scattering. To elucidate interaction mechanism of Ru NPs with different moieties in carbon substrate, we calculated the average formation energies of three simulated structures (i.e., Ru/C, Ru/N–C and Ru/Fe–N–C). HAADF‐STEM image of Ru/Fe–N–C estimates the average size of Ru NPs is around 1.8 nm, and the average Ru–Ru coordinate is around 4. Combining with the Ru NPs information from the previous studies,^[^ ^33^ ^]^ we built a Ru_6_ NP in octahedron configuration with adjacent Fe atoms (Figure 1j). The theoretical model was used to fit Ru/Fe–N–C EXAFS again (Figure S13, Supporting Information), which shows more reasonable fitting results with four‐coordinated Ru–N (Table S3, Supporting Information). The new fitting results imply that the Ru–Ru coordination ratio between Ru/N–C and Ru/Fe–N–C is still 2.17. Applying the same strategy in our previous study,^[^ ^26^ ^]^ we use this ratio to scale up the size of NPs in Ru/N–C, and the result matches well with the size of Ru_13_ with dodecahedron structure reported before.^[^ ^33^ ^]^ Based on the formula of Δ*E* ^atom^ = (*E* ^atom‐NCS^ – *N* ^atom^E^atom^)/*N* ^atom^, as shown in Figure 1j and Table S5 (Supporting Information), the Ru_6_ octahedron configuration on Fe–N_4_ carbon substrate is preferentially formed with the lowest value for average formation energy. Both the experimental and theoretical results show that the smaller metal NPs thermodynamically prefer to form on the Fe–N_4_ substrate.

*Electrochemical HER Performance*: To investigate the effect of the Ru SAs and Ru NPs on the HER activity, the performance of Ru/Fe–N–C and the control samples was firstly evaluated in 1 m KOH electrolyte by a three‐electrode electrochemical cell. The HER activity was normally evaluated by the overpotential (*η*_10_) versus reversible hydrogen electrode (RHE) at a current density of 10 mA cm^−2^, and it is the current density for an expected 12.3% solar water‐splitting conversion efficiency.^[^ ^34^ ^]^ As shown in **Figure** **2**a, no evident cathodic current is observed for pure Fe–N–C. However, the synergy of Ru with the Fe–N–C matrix shows an outstanding HER activity with a very small onset potential at the thermodynamic potential (i.e., 0 V), demonstrating that Ru is indispensable in boosting the HER activity. It exhibits a very low *η*_10_ value of ≈9 mV, even 25 mV smaller than commercial Pt/C (≈34 mV). Normalized to respective loading, Ru/Fe–N–C shows a large mass activity (−2.56 A ${\mathrm{mg}}_{\mathrm{Ru}}^{-1}$), which is 3.3 times higher than commercial Pt/C (−0.78 A ${\mathrm{mg}}_{\mathrm{Pt}}^{-1}$) at 50 mV (Figure S20, Supporting Information). To highlight the key role of Fe and N in HER, N–Fe‐free Ru/C and Fe‐free Ru/N–C were also prepared (see the Supporting Information), both of which possess similar pore textures as Ru/Fe–N–C (Figure S2, Supporting Information). Although N‐doping leads to a smaller *η*_10_ value (64 mV) for Ru/N–C than 85 mV for Ru/C, both of them are far larger than 9 mV for Ru/Fe–N–C, as shown in the obvious negative shift of the polarization curves (Figure 2a). These results reveal the significant roles of both Ru SAs and Ru NPs in improving the HER activity of Ru/Fe–N–C. Notably, the HER activity of present Pt/C is among the best in the previous reports,^[^ ^14^, ^17^, ^19^ ^]^ which indicates the excellent intrinsic activity for Ru/Fe–N–C, not resulting from the use of a poor Pt/C reference.

**Figure 2:**
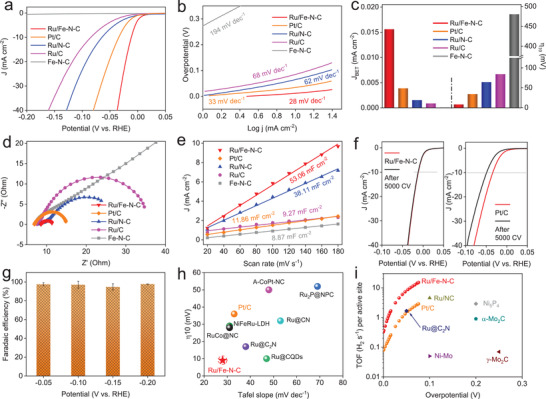
Hydrogen evolution performance of Ru/Fe–N–C and the control samples. a) *iR*‐corrected polarization curves with a scan rate of 2 mV s^−1^ in 1 m KOH solution. b) Tafel plots from the polarization curves. c) The comparison of normalized current densities based on BET surface area at −0.05 V (vs RHE) and overpotential at 10 mA cm^−2^ (*η*_10_). d) The Nyquist plots at −0.1 V (vs RHE). e) The electrochemical double layer capacitance (*C*_dl_) of Ru/Fe–N–C and other catalysts. f) Durability test of Ru/Fe–N–C and Pt/C by recording the polarization curves before and after 5000 cycles. g) Faradaic efficiency of Ru/Fe–N–C at different applied potentials. h) The *η*_10_ versus Tafel slope of Ru/Fe–N–C in contrast to the reported catalysts. i) TOF values of Ru/Fe–N–C compared with the reported catalysts.

Tafel slope reflects the interfacial kinetics, and HER involves either the Volmer–Heyrovsky or the Volmer–Tafel mechanism.^[^ ^18^, ^35^, ^36^, ^37^ ^]^ The doping of active Fe–N sites into substrate results in a significant decrease of Tafel slope from 62 and 68 mV dec^−1^ for Ru/N–C and Ru/C to 28 mV dec^−1^ for Ru/Fe–N–C, respectively. The value is even 5 mV dec^−1^ smaller than that of Pt/C (Figure 2b), suggesting a Volmer–Tafel mechanism for Ru/Fe–N–C. The exchange current density (*J*_0_) was obtained by extrapolating the Tafel plots. As shown in Figure S21 (Supporting Information), Ru/Fe–N–C possesses a *J*_0_ of 1.94 mA cm^−2^, which is much higher than other contrast catalysts, and even surpasses Pt/C. Moreover, Ru/Fe–N–C gives the highest specific current density of 0.0156 mA cm^−2^ (−0.05 V vs RHE), which is 4, 10.4, 18.1, 312 times higher than Pt/C (0.0039 mA cm^−2^), Ru/N–C (0.0015 mA cm^−2^), Ru/C (0.0086 mA cm^−2^), Fe–N–C (0.00005 mA cm^−2^), respectively (Figure 2c). Electrochemical impedance spectroscopy (EIS, −0.1 V vs RHE) of Ru/Fe–N–C exhibits the smallest semicircle, which indicates the intrinsic fast charge transfer at the interface of electrocatalyst and electrolyte (Figure 2d). These results demonstrate that the HER kinetics are sharply enhanced by anchoring both Ru SAs and NPs on Fe–N–C substrate.

The electrochemical double‐layer capacitance (*C*_dl_) is another effective technique to estimate the electrochemically active surface area (ECSA) of samples with similar structures and compositions.^[^ ^38^, ^39^ ^]^ The *C*_dl_ values were obtained by use of cyclic voltammetry versus scan rates (Figure S22, Supporting Information). As depicted in Figure 2e, the *C*_dl_ value follows the order as Fe–N–C (8.87 mF cm^−2^) < Ru/C (9.27 mF cm^−2^) < Ru/N–C (38.11 mF cm^−2^) < Ru/Fe–N–C (53.06 mF cm^−2^). The larger *C*_dl_, the better proton exchangeability between active sites and electrolyte. Thus, the above results show the optimized chemical composition of Ru/Fe–N–C for HER with enhanced activity when compared to Ru/C and Ru/N–C. The Ru/Fe–N–C exhibits a neglectable increase of *η*_10_ after 5000 CV tests, while commercial Pt/C shows a larger degradation of ≈15 mV under the similar condition (Figure 2f). And the HRTEM characterizations confirm that the morphology has no evident change after long‐term operation (Figure S23, Supporting Information), revealing the remarkable stability of Ru/Fe–N–C. This is possibly attributed to strong coupling of Ru and Fe–N–C matrix, that keeping it from reconstruction. The Ru/Fe–N–C also exhibits high durability of oxidation‐resistance, and the valence state of Ru remains unchanged after long‐term exposure to air (Figure S24, Supporting Information). Subsequently, gas chromatography was employed to detect the H_2_ production, which shows that the Faradaic efficiency of Ru/Fe–N–C is nearly 100% under a wide range of potentials (Figure 2g; Figure S25, Supporting Information). In terms of *η*_10_ (9 mV) and Tafel slope (28 mV dec^−1^), Ru/Fe–N–C outperforms or is comparable to the state‐of‐the‐art metal‐based HER electrocatalysts including NiFeRu‐LDH (29 mV, 31 mV dec^−1^),^[^ ^40^ ^]^ A–CoPt–NC (50 mV, 48 mV dec^−1^),^[^ ^41^ ^]^ Ru@C_2_N (17 mV, 38 mV dec^−1^),^[^ ^4^ ^]^ Ru_2_P/NPC (52 mV, 69 mV dec^−1^),^[^ ^42^ ^]^ Ru@CQDs (10 mV, 47 mV dec^−1^),^[^ ^19^ ^]^ RuCo@NC (28 mV, 31 mV dec^−1^),^[^ ^14^ ^]^ Ru@CN (32 mV, 53 mV dec^−1^),^[^ ^17^ ^]^ RuSAs + RuNPs@MHC (7 mV, 29 mV dec^−1^),^[^ ^43^ ^]^ and Cu/Ru@G_N_ (8 mV, 29 mV dec^−1^)^[^ ^44^ ^]^ (Figure 2h; Table S6, Supporting Information). Turnover frequency (TOF) is the most effective figure of merit to characterize intrinsic electrocatalytic activity of catalysts. The number of active sites for Ru/Fe–N–C and Pt/C were estimated by means of Cu underpotential deposition (UPD) (Figure S26, Supporting Information). As illustrated in Figure 2i and Table S7 (Supporting Information), Ru/Fe–N–C gives a TOF value of 3.6 and 8.9 H_2_ s^−1^ at an overpotential of 25 and 50 mV, respectively, which is 7.6 and 6.1 times larger than that of Pt/C (0.47 H_2_ s^−1^ at 25 mV overpotential and 1.46 H_2_ s^−1^ at 50 mV overpotential). In addition, the TOF value of Ru/Fe–N–C significantly exceeds those of Ru‐based catalysts, such as Ru@C_2_N (0.76 H_2_ s^−1^ at 25 mV overpotential; 1.66 H_2_ s^−1^ at 50 mV overpotential),^[^ ^4^ ^]^ Ru/NC (4.55 H_2_ s^−1^ at 100 mV overpotential),^[^ ^21^ ^]^ and is also superior than those of *α*‐Mo_2_C (0.9 H_2_ s^−1^ at 200 mV overpotential),^[^ ^45^ ^]^ *γ*‐Mo_2_N (0.07 H_2_ s^−1^ at 250 mV overpotential),^[^ ^45^ ^]^ Ni_5_P_4_ (2.9 H_2_ s^−1^ at 200 mV overpotential),^[^ ^46^ ^]^ Ni‐Mo (0.05 H_2_ s^−1^ at 100 mV overpotential).^[^ ^47^ ^]^

*Effect of Substrates*: In order to further understand the origin of high HER activity of Ru/Fe–N–C with both Ru SAs and Ru NPs, the influence of various substrates on Ru moieties was investigated in detail by X‐ray photoelectron spectroscopy (XPS), X‐ray absorption spectroscopy (XAS) and DFT calculations. Ru 3p XPS core‐level spectra show that the peaks at ≈462.2 and ≈484.4 eV are allocated to the Ru^0^ moiety and the other peaks are Ru*^n^* ^+^ in three samples (**Figure** **3**a). Ru/Fe–N–C has the highest content of Ru*^n^* ^+^ (≈32%), which can be attributed to the strong interaction between Ru NPs and Fe–N–C substrate, as well as the high density of Ru SAs coordinated with C/N groups (Figures 1e and 3b). The Fe2p exhibits a negative shift by ≈0.6 eV after Ru loading (Figure S29, Supporting Information), implying the increased electron transfer of Ru NPs to the Fe–N–C substrate. The identical result is attained in the XAS measurement as well. The negative shift to lower energy region is shown in the Fe K‐edge XANES spectra when Ru species were incorporated, suggesting a more reduced valence state of Fe in Ru/Fe–N–C than that in pure Fe–N–C (Figure 3c). The Ru K‐edge XANES curves reveal the absorption edge of Ru/Fe–N–C is higher than Ru/N–C, and a special valence state of +2.4 is obtained for Ru in Ru/Fe–N–C sample (Figure 3d–e). Associated with the Ru EXAFS results (Figure 1e), such a high valence state of Ru in Ru/Fe–N–C may derive from the synergetic effect of Ru SAs and small‐sized Ru NPs. The well consistence of XPS and XANES effectively confirms the strong electronic interaction between Ru and Fe–N–C support, and it may account for the fact of ultrahigh HER activity. To further identify the charge transfer between Ru NPs and various carbon substrates, we also calculated the Bader charge of Ru/C, Ru/N–C and Ru/Fe–N–C system. From the quantitative (Figure 3f; Table S5, Supporting Information) and qualitative analysis (Figure 3g–i) of charge transfer based on DFT calculation, it is found that Fe–N–C substrate prefers to regulating the electron structure of Ru NPs with the largest amount of charge transfer when comparing with other substrates.

**Figure 3:**
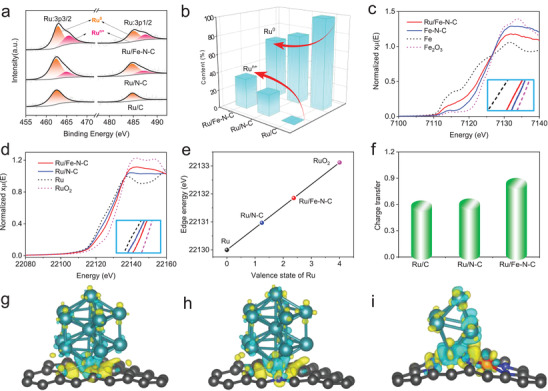
The interaction between Ru species and substrate in Ru/C, Ru/N–C, and Ru/Fe–N–C. a,b) High‐resolution Ru 3p XPS spectra a) and the corresponding Ru^0^ and Ru*^n^* ^+^ content b) of Ru/Fe–N–C, Ru/N–C, and Ru/C. c) Fe K‐edge XANES spectra of Fe–N–C, Ru/Fe–N–C, Fe, and Fe_2_O_3_. d) Ru K‐edge XANES spectra of Ru/Fe–N–C, Ru/N–C, Ru, and RuO_2_. e) Valence state of Ru in Ru/Fe–N–C and Ru/N–C, calculated from XANES results. f) The charge transfer between Ru nanoclusters and substrate in three calculated structures (Ru/C, Ru/N–C, and Ru/Fe–N–C). g–i) The charge‐density differences of three predicted structures (Ru/C, Ru/N–C, and Ru/Fe–N–C) by DFT calculation. Yellow and blue regions represent the accumulation of positive and negative charge, respectively.

*Understanding the Origin of High Activity by DFT Calculation*: To understand catalytic role of multiple active sites and construct a unified picture, density functional theory (DFT) calculations were performed to investigate catalytic sites and corresponding energetics of Ru/C, Ru/N–C, and Ru/Fe–N–C. The hydrogen adsorption free energy Δ*G*_H*_ is an effective descriptor to determine the HER activity, while water dissociation barrier of catalysts is considered as an important parameter to estimate the catalytic activity.^[^ ^48^ ^]^ Since Ru/Fe–N–C exhibits highly active catalysis by experiment, different atomic sites of Ru cluster in Ru/Fe–N–C were used to calculate Δ*G*_H*_ (**Figure** **4**a). The Ru atoms located from faraway to connecting with Fe–N–C exhibit an increasing hydrogen adsorption energy, Δ*G*_H*_(Ru1) = 0.025 eV and Δ*G*_H*_(Ru3) = −0.403 eV. The optimal HER active site is considered as Ru1 (Figure 4b). For comparison, we also calculated Δ*G*_H*_ of Ru_6_ nanoparticle without substrate with the strong hydrogen binding energy (−0.37 eV), which is unfavorable for hydrogen desorption. In general, the different catalytic activity is attributed to oxidization degree of different atoms of Ru NP on substrate. The Bader charge calculations show about 0.989 e^−^ charge transfer from Ru NPs to Fe–N_4_ entity. In comparison, the atoms connected with Fe–N–C substrate have stronger charge transfer than those faraway Ru atoms. The structure–property relationship is also exhibited in the various atoms of Ru/N–C and Ru/C system (Figure S30, Supporting Information).

**Figure 4:**
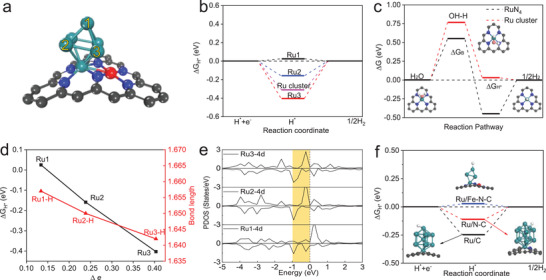
DFT calculations. a) Atomic configurations of simulated Ru cluster with numbered Ru atoms in Ru/Fe–N–C system. b) Hydrogen adsorption free energies (Δ*G*_H*_) of possible sites. The Ru1 site of interface between Ru cluster and Fe–N–C substrate exhibits high activity for HER. c) Gibbs free energy diagram of HER on Ru–N_4_ and Ru cluster in Ru/Fe–N–C system including reactant initial state, intermediate state, final state, and an additional transition state representing water dissociation. Δ*G*_B_ indicates water dissociation free energy barrier. d) Correlation between and among Δ*G*_H*_, Ru—H bond length and charge transfer Δ*e* ^−^ of various Ru sites in simulated Ru/Fe–N–C with adsorbed H atom. e) The projected density of state (pDOS) of various Ru sites of Ru cluster in Ru/Fe–N–C system. f) Comparison of Δ*G*_H*_ of HER on Ru/Fe–N–C, Ru/N–C, Ru/N–C system.

It is widely accepted that hydrogen evolution in alkaline contains two continuous steps of water dissociation and hydrogen desorption. Besides hydrogen desorption, the barrier height (Δ*G*_B_) of water dissociation also plays an important role in determining overall alkaline HER reaction kinetic rate. Based on our DFT calculations, it is found that two active sites of Ru–N_4_ and Ru3 of Ru cluster in Ru/Fe–N–C system exhibit much lower activation barriers (0.550 and 0.774 eV) for water dissociation, respectively, than Pt catalysis (0.94 eV).^[^ ^18^ ^]^ From the kinetic viewpoints, atom‐dispersed Ru–N_4_ could accelerate water dissociation to provide neutral hydrogen source. Furthermore, the atom‐dispersed Ru also has appropriate hydrogen binding energy. Thus, the atom‐dispersed Ru is of much importance to high‐efficiency HER in alkaline media (Figure 4c and Figures S31–32, Supporting Information).

Higher hydrogen binding energy corresponds to higher activity of water dissociation, but lower hydrogen desorption capacity. As shown in Figure 4d, there is a linear correlation between and among Δ*G*_H*_, Ru—H bond length and the amount of charge transfer Δ*e* ^−^, indicating the more active electron transfer and the stronger hydrogen binding energy. The similar correlation is further verified by the relationship between the Ru—H bond length and charge transfer Δ*e* ^−^. Furthermore, we also studied the projected density of state (pDOS) of various Ru sites in Ru/Fe–N–C system to understand the origin of high activity (Figure 4e). Through comparing Ru1‐4d, Ru2‐4d, and Ru3‐4d active electron density near Fermi (highlighted by yellow rectangular areas), it is observed that the amounts of electron states 4d orbitals of Ru1–3 atom between −1 and 0 eV gradually increase corresponding to the intensity of hydrogen binding from weak to strong. The pDOS before and after H absorbed of Ru/C and Ru/N–C system are illustrated in Figure S33 (Supporting Information). Moreover, compared to Δ*G*_H*_ of Ru/C and Ru/N–C system (Figure 4f), the Fe–N–C substrate prefers to regulate the intrinsic charge distribution of Ru NPs, further optimizing the HER performance.

In summary, we prepared an efficient hydrogen evolution catalyst by combining Ru SAs with Ru NPs on the Fe/N codoped carbon substrate (Fe–N–C). The resulting Ru/Fe–N–C catalyst exhibits markedly enhanced reaction kinetics, large mass and BET surface area activity, as well as high intrinsic activity (TOF) for HER. Theoretical calculations suggest that the single atom Ru–N_4_ moieties could significantly improve the water dissociation kinetics, while the Ru NPs are beneficial to hydrogen evolution. We found that the incorporation of Fe species could promote Ru NPs into isolated Ru atoms and small‐sized Ru NPs. Moreover, the Fe–N–C substrate could further adjust the charge distribution of Ru NPs, thus optimizing the hydrogen adsorption energy. This study demonstrates the potential of special substrate in modifying particle size and electronic structure of metal NPs, paving a new avenue for designing efficient electrocatalysts in energy conversion and storage.

## Experimental Section

##### Chemical Reagents

All chemicals, including ruthenium chloride hydrate (Aladdin, 35.0–42.0 wt% Ru basis), d (+)‐glucose monohydrate (Sinopharm Chemical Reagent Co., Ltd.), dicyandiamide (Aladdin, 99%), Ludox HS40 colloidal silica (Aldrich, 40 wt%), iron chloride anhydrous (Sinopharm Chemical Reagent Co., Ltd.), potassium hydroxide (Sinopharm Chemical Reagent Co., Ltd., ≥85%), sodium hydroxide (Sinopharm Chemical Reagent Co., Ltd.), potassium phosphate monobasic (Aladdin, ≥99%), Nafion solution (Sigma‐Aldrich, 5 wt%), Pt/C catalyst (Johnson Matthey, 40 wt%), and concentrated sulfuric acid (Sinopharm Chemical Reagent Co., Ltd, 95–98%), were used as received without further purification.

##### Materials Synthesis

The Ru nanoparticles anchored onto a Fe–N–C support (Ru/Fe–N–C) with uniform mesopores were prepared via a pyrolysis and subsequent etching strategy. Typically, a certain amount of glucose (2 g), dicyandiamide (2 g), iron chloride anhydrous (0.3 g) and 8 g colloid silica solution were mixed with 50 mL deionized water under vigorously stirring to get a homogeneous mixed solution, followed by the addition of 20 mL ruthenium chloride aqueous solution (0.048 × 10^−3^ m). After stirring for ≈30 min, the mixture was evaporated by heating up to 110 °C and maintained at this temperature under continuously stirring. The dried brown product was pyrolyzed at 800 °C for 2 h in quartz tube furnace under Ar atmosphere with a heating rate of 5 °C min^−1^. After cooling, the silica template was etched off with 2 m NaOH solution. After being rinsed several times with deionized water and ethanol, the black solid was further leached in 0.5 m H_2_SO_4_ at 60 °C for 2 h to remove the unstable iron‐containing species. Finally, the catalyst was collected by centrifugation and purified by deionized water and ethanol for several times, and then dried under oven at 90 °C. The resultant dark solid, named as Ru/Fe–N–C, was ground into a fine powder for further analyses. The control samples of Fe single atom coordinated with pyridinic‐N‐doped carbon framework (Fe–N–C), Ru nanoparticles supported on carbon (Ru/C), and Ru nanoparticles dispersed within N‐doped carbon matrix (Ru/N–C) were prepared using the same process. Specifically, Fe–N–C was prepared without the addition of Ru source, Ru/C was prepared in the absence of Ru, Fe, and N sources, and Ru/N–C was synthesized without adding Fe source. Other samples possess diverse ruthenium content were labeled as Rux/Fe–N–C (*x* = 0.05, 0.1, 0.3).

##### Structural Characterization

A JEOL S‐4800 SEM was used to characterize the sample morphology. Samples for analysis were mounted onto a conductive carbon double‐sided sticky tape. TEM measurement was performed employing a JEOL JEM‐2100F microscope operating at an accelerating voltage of 200 kV. Samples were deposited on a thin amorphous porous carbon film supported by copper grid derived from ultrasonic ethanol solutions. High‐resolution TEM (HRTEM) was performed on JEM‐ARM300F at an acceleration voltage of 300 kV with an EDS attachment. The XRD patterns were collected on a Bruker D8 ADVANCE diffraction workstation with Cu K*α* radiation. Raman spectroscopy were performed using a DXR Raman Microscope (Thermal Scientific Co., USA) with 532 nm excitation wavelength. Nitrogen adsorption–desorption measurements were conducted at −196 °C on a Quadrasorb SI surface area and pore sizes analyzer (Quantachrome Ins). The specific surface area and pore sizes were calculated based on the Brunauer–Emmett–Teller (BET) and Barrett–Joyner–Halenda (BJH) methods, respectively. All samples were dehydrated under vacuum at 200 °C overnight before each measurement. XPS characterizations were carried out on an ESCALAB 250Xi X‐ray photoelectron spectrometer (Thermal Scientific Co., USA) with Al K*α* radiation. The elemental spectra were all calibrated with respect to C1s peaks at 284.8 eV. The Ru metal content of the catalysts was measured by inductively coupled plasma atomic emission spectroscopy (ICP‐AES). A certain amount of sample was mixed with 5 mL nitric acid, which was transferred to high pressure digestion tank, sealed and remained at 235 °C for 10 h. Subsequently, 5 mL hydrochloric acid was added in above mixture, heating until the sample is completely dissolved. And the resulting solution was examined by using iCAP 6300 spectrometer. X‐ray absorption fine‐structure spectroscopy (XAFS) was performed the Advanced Photo Source at Argonne National Laboratory at the 5‐BM beamline. The nanoparticle samples were drop cast onto Kapton tape and measured from 150 eV below the K‐edge absorption of Fe (7.11 keV) or Ru (22.10 keV) to 800 eV above the respective absorption edges. Metal foils of either Fe or Ru were used to calibrate *E*_0_ and served as reference material for subsequent linear combination fitting of the X‐ray absorption near‐edge structure (XANES). All data processing and linear combination fitting were performed using the software program Athena.

##### Electrochemical Measurements

The electrochemical measurements were carried out in a three‐electrode setup using a CHI 760C workstation at room temperature. To prepare the working electrode, 5 mg electrocatalyst and 25 µL Nafion solution were dispersed in 500 µL of 1:1 (v:v) water/ethanol by sonication to form a homogeneous ink. Then, 10 µL suspension was loaded onto a 5 mm diameter polished glassy carbon electrode (catalyst loading amount ≈0.485 mg cm^−2^). A graphite rod and saturated calomel electrode (SCE) were used as the counter electrode and reference electrode, respectively. The reference electrode was experimentally calibrated against RHE. Linear sweep voltammetry (LSV) was conducted in 1.0 m aqueous KOH with a scan rate of 2 mV s^−1^ and a rotation speed of 1600 rpm. Commercial 20 wt% Pt/C was used as a reference to evaluate the electrocatalytic performance of as‐prepared catalysts. The cyclic voltammetry (CV) measurements were conducted at 1600 rpm with a sweep rate of 100 mV s^−1^ for 5000 times to investigate the cycling stability.

##### Calculation of the Turnover Frequency (TOF)

The number of active sites (*n*) was qualified by using the copper underpotential deposition (Cu UPD) with the following equation^[^ ^4^ ^]^

(1) $$\begin{array}{c} n=\frac{Q_{\mathrm{Cu}}}{2F}\; \end{array}$$

where *Q*_Cu_ is the copper stripping charge, and *F* is the faraday constant (96 485 C mol^−1^).

The TOF was calculated with the following equation

(2) $$\begin{array}{c} \mathrm{TOF}=\frac{I}{2Fn} \end{array}$$

The factor 1/2 is based on the consideration that two electrons are required to form one hydrogen molecule.

##### Faradaic Efficiency Measurements

Faradaic efficiency (FE) of Ru/Fe–N–C was measured at different potentials (−0.05, −0.10, −0.15, −0.20 V vs RHE) by gas chromatography (7820A, Agilent), and a thermal conductivity detector (TCD) was used for H_2_ quantification. In a custom‐made two compartment cell (single cell: 50 mL) separated by a Nafion 117 membrane, each compartment of the cell was filled with 35 mL 1.0 m KOH. 20 µL suspension was droped onto a 1 × 1 cm^2^ diameter carbon cloth electrode (catalyst loading amount ≈0.97 mg cm^−2^). The H_2_ gas was purged out from the cell by using 1 mL syringe and injected into GC. FE was calculated according to following relationship^[^ ^49^ ^]^

(3) $$\begin{array}{c} \mathrm{FE}=\frac{2F\cdot n_{H_{2}}}{Q}\; \end{array}$$

where is $n_{H_{2}}$ is the amount of hydrogen (mol), and *Q* is the total amount of charge passed through the cell (C).

##### Theoretical Calculations

Spin‐polarized density functional theory (DFT) calculations were performed using the Vienna Ab initio Simulation Package (VASP) plane‐wave DFT code, with the generalized gradient approximation of Perdew–Burke–Ernzerhof to describe electron exchange and correlation.^[^ ^50^ ^]^ The plane‐wave basis is cut off by 500 eV.^[^ ^51^ ^]^ The projector‐augmented plane wave (PAW) was used to describe the electron–ion interactions.^[^ ^52^ ^]^ A set of (3 × 3 × 1) *k*‐points were carried out for geometric optimization, and the convergence threshold was set as 10^−4^ eV in energy and 0.05 eV Å^−1^ in force, respectively. The Hubbard‐type U correction for the strong‐correlation d‐electrons of transition metals is taken into account.^[^ ^53^ ^]^ To calculate transition barriers, it was performed climbing image nudge elastic band calculations^[^ ^54^ ^]^ on each of these combinations of the final H+OH configuration with the most stable initial H_2_O configuration and selected the combination with the least energy barrier for each surface.

For the systems, the free energy of the adsorbed state is calculated as

(4) $$\begin{array}{c} \Delta G_{H*}=\Delta E_{H*}+\Delta E_{\mathrm{ZPE}}-T\Delta S \end{array}$$

where Δ*E*_H*_ is the hydrogen chemisorption energy, and Δ*E*_ZPE_ is the difference corresponding to the zero point energy between the adsorbed state and the gas phase. As the vibration entropy of H* in the adsorbed state is small, the entropy of adsorption of 1/2 H_2_ is Δ*S*_H_ ≈ −1/2$S_{H_{2}}^{0}$, where $S_{H_{2}}^{0}$ is the entropy of H_2_ in the gas phase at the standard conditions.

## Conflict of Interest

The authors declare no conflict of interest.

## Supporting information

Supporting Information
