# Supplementary material for: Partial‐Single‐Atom, Partial‐Nanoparticle Composites Enhance Water Dissociation for Hydrogen Evolution
Source: Adv Sci (Weinh). 2020 Nov 25;8(2):2001881. doi: 10.1002/advs.202001881 (PMC7816713; doi:10.1002/advs.202001881)
Supplement: Supplementary file 1 — Supporting Information [file ADVS-8-2001881-s001.pdf]

WILEY-VCH

© 2020 Wiley-VCH GmbH

ADVANCED SCIENCE
Open Access

Supporting Information

for *Adv. Sci.*, DOI: 10.1002/advs.202001881

Partial-Single-Atom, Partial-Nanoparticle Composites Enhance Water Dissociation for Hydrogen Evolution

Chun Hu, Erhong Song, Maoyu Wang, Wei Chen, Fuqiang Huang, Zhenxing Feng, Jianjun Liu*, and Jiacheng Wang**

Supporting Information

Partial-Single-Atom, Partial-Nanoparticle Composites Enhance Water Dissociation for Hydrogen Evolution

Chun Hu, Erhong Song, Maoyu Wang, Wei Chen, Fuqiang Huang, Zhenxing Feng, Jianjun Liu*, and Jiacheng Wang**

Supporting Data

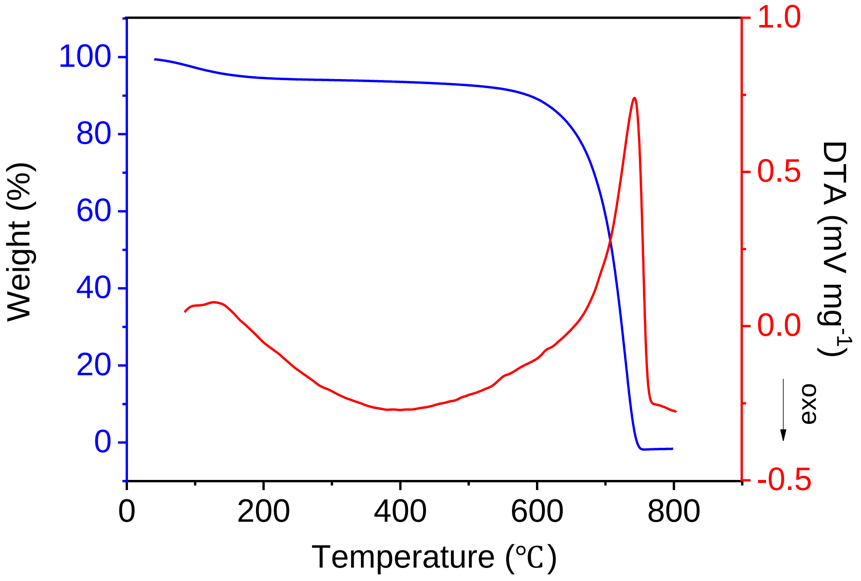

Figure S1 TG-DTA curves of pure g-C_3N_4 obtained in a nitrogen flow (10 °C/min). Pristine g-C_3N_4 shows 100% weight loss at 750 °C in a nitrogen flow with a ramp of 10 °C/min, implying its complete decomposition.

Ru/Fe-N-C sample was prepared from glucose, dicyanamide, $FeCl_3$, $RuCl_3$, and colloidal silica *via* pyrolysis and calcination (details in the experimental section). During pyrolysis, the as-formed layered graphitic carbon nitride (g-C_3N_4) composed of repeated melem building units could act as nitrogen source and confined nano-space for the formation of single-atom Fe-N_4 sites, that could *in-situ* intergrate with resulting Ru NPs. And the redundant g-C_3N_4 was naturally decomposed at increased pyrolysis temperature of 800 °C (Figure S1). Additionally, it is noteworthy that the presence of mesopores makes the maximized utilization of Fe-N_4 moieties by means of forming channels to the buried sites.[1] The subsequent etching treatment by

alkaline and acid solutions removed silica template and unstable Fe-related NPs to obtain black Ru/Fe-N-C. During the calcination, Fe atom tends to coordinate with N species, obtaining Fe-N_4 moieties. Moreover, the layered texture of g-C_3N_4 and strong achoring ability of electronegative N atoms are able to avoid the overgrowth of Ru NPs.

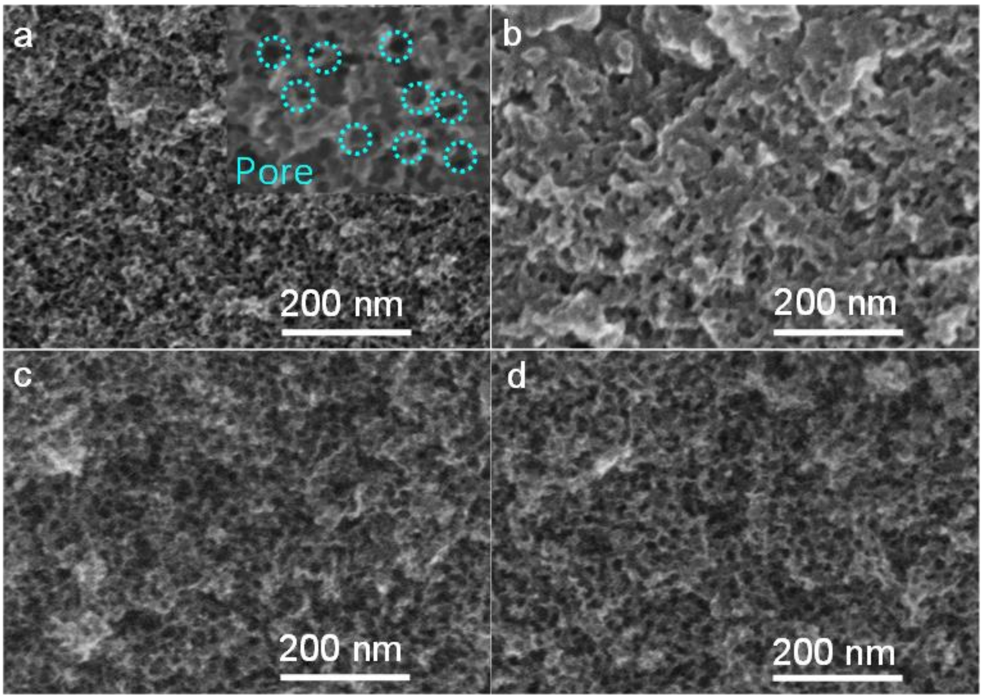

Figure S2 The scanning electron microscope (SEM) images of Ru/Fe-N-C, Fe-N-C, Ru/N-C and Ru/C.

The scanning electron microscopy (SEM) images shows that Ru/Fe-N-C, Fe-N-C, Ru/N-C and Ru/C possess uniform porous textures with abundant voids originated from residual room after removing silica (Figure S2). Well-defined porosity could increase the exposure of the accessible catalytic sites, thus improving the activity.

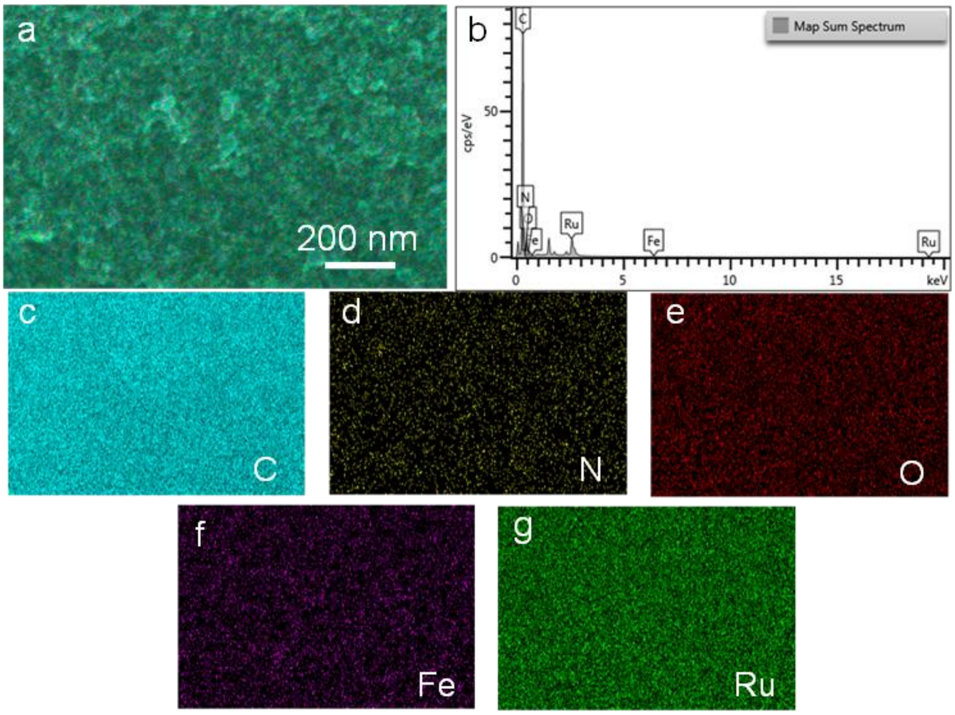

Figure S3 (a) Low magnification SEM image with the corresponding elemental mapping images of C (c), N (d), O (e), Fe (f) and Ru (g) for Ru/Fe-N-C. The resulting Ru/Fe-N-C consists of C, N, Fe, and Ru elements, as well as small amount of O element derived from trapped moisture and/or edged oxygen-containing groups.

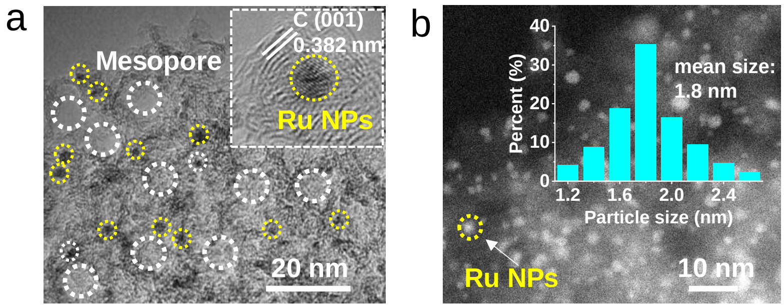

Figure S4 (a) Transmission electron microscopy (TEM) of Ru/Fe-N-C (Dashed yellow circles indicate Ru NPs, while white ones show the mesopores derived from residual room by etching off silica NPs) and high-resolution TEM (HRTEM) (inset). (b) High-angle annular dark-field scanning TEM (HAADF-STEM) image (inset: particle-size distribution of Ru NPs).

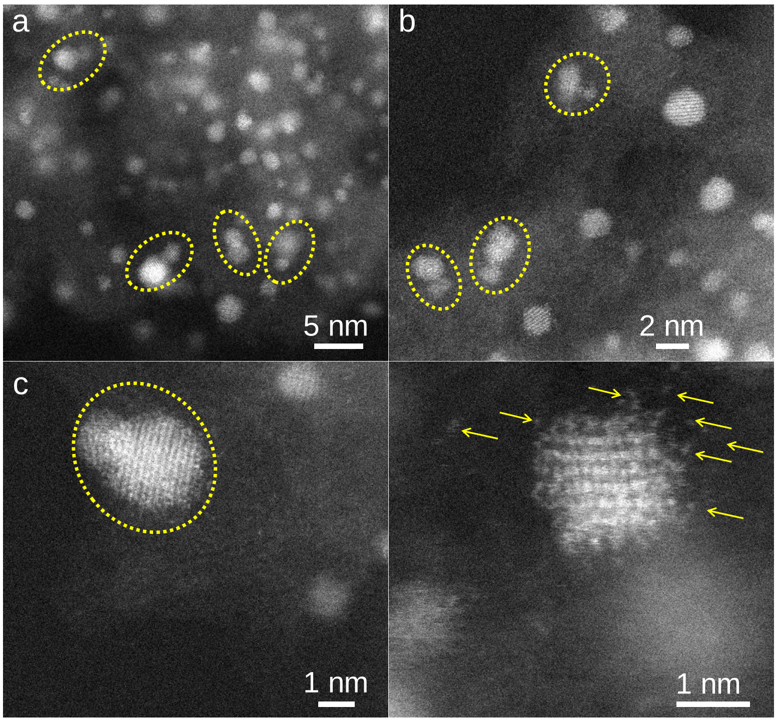

Figure S5 The High-angle annular dark-field scanning TEM (HAADF-STEM) images of Ru/Fe-N-C, where the dashed yellow circles indicate the aggregation of nanoparticles, and the yellow arrows signify Ru single-atoms/nanoclusters.

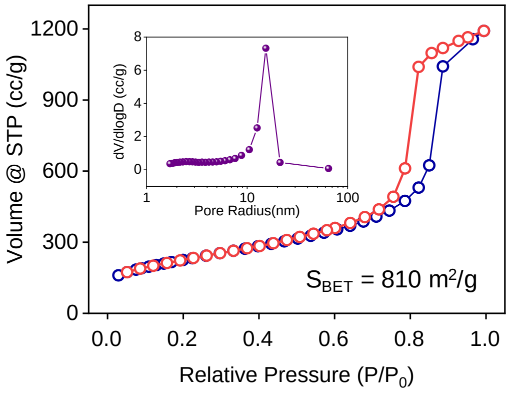

Figure S6 N_2 adsorption-desorption isotherms (inset: pore size distribution from the adsorption branch).

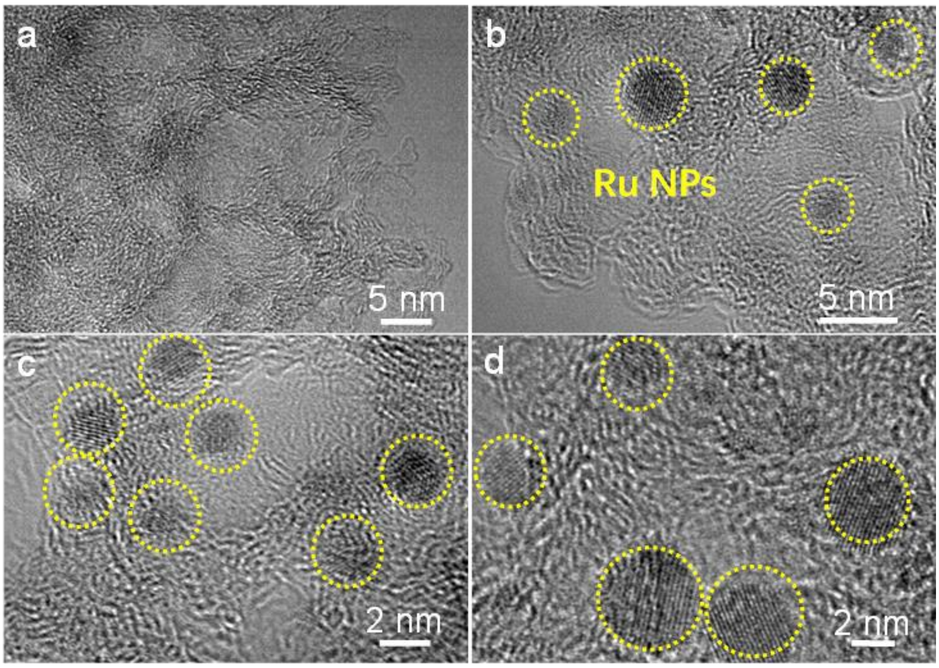

Figure S7 The high-resolution transmission electron microscopic (HRTEM) images of Fe-N-C, Ru0.05/Fe-N-C, Ru0.1/Fe-N-C, Ru0.3/Fe-N-C. With the increasing of Ru addition, the particle size of Ru tends to become larger.

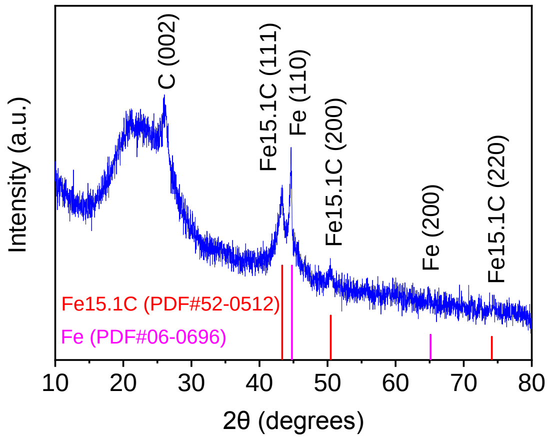

Figure S8 The XRD pattern of Ru/Fe-N-C before acid etching. It clearly indicates that Fe particles exist in the nanocomposite obtained by pyrolysis of glucose, dicyanamide, $FeCl_3$, and $RuCl_3$.

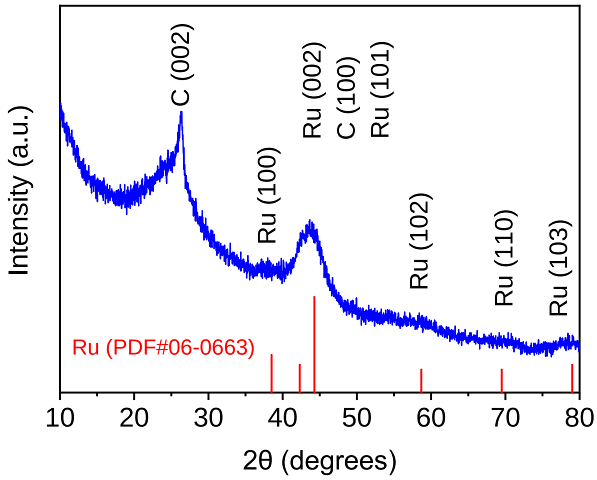

Figure S9 The X-ray diffraction (XRD) pattern of Ru/Fe-N-C.

The X-ray diffraction (XRD) pattern of Ru/Fe-N-C reveals well removal of Fe-derived species after acid etching (Figure S8-9).

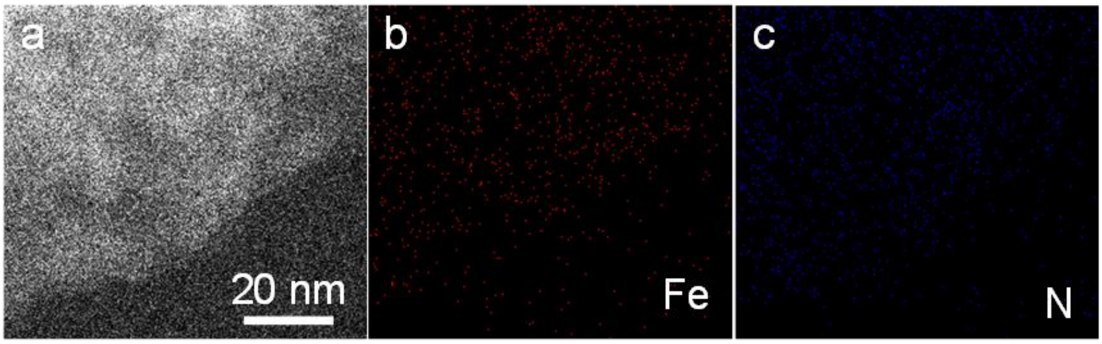

Figure S10 The high-angle annular dark-field scanning transmission electron microscopy (HAADF-STEM) of pristine Fe-N-C without Ru loading (a) and the corresponding elemental mappings of (b) Fe (red) and (c) N (blue). The Fe and N contents are 0.86 and 13.46 at%, respectively. No Fe nanoparticles could be observed, showing the high dispersion of Fe elements in the Fe-N-C sample.

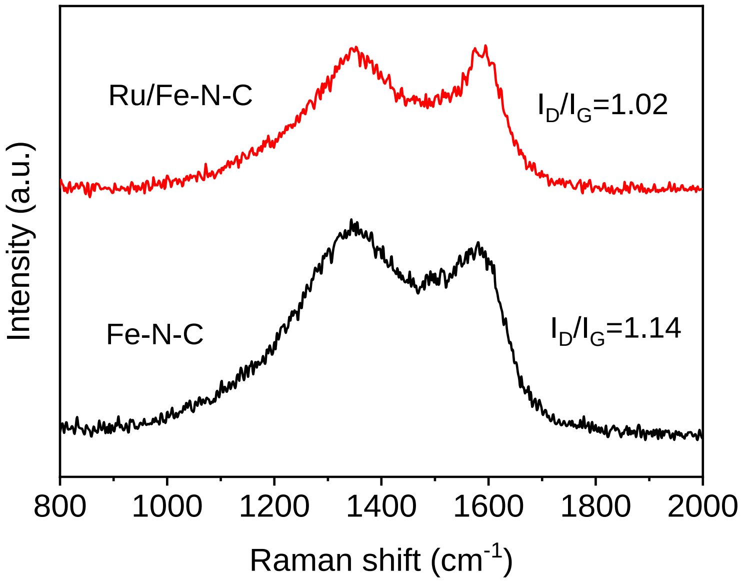

Figure S11 Raman spectra of Fe-N-C and Ru/Fe-N-C. A lower I_D/I_G for Ru/Fe-N-C value suggests the increased graphitization, which is advantageous for electron transport.

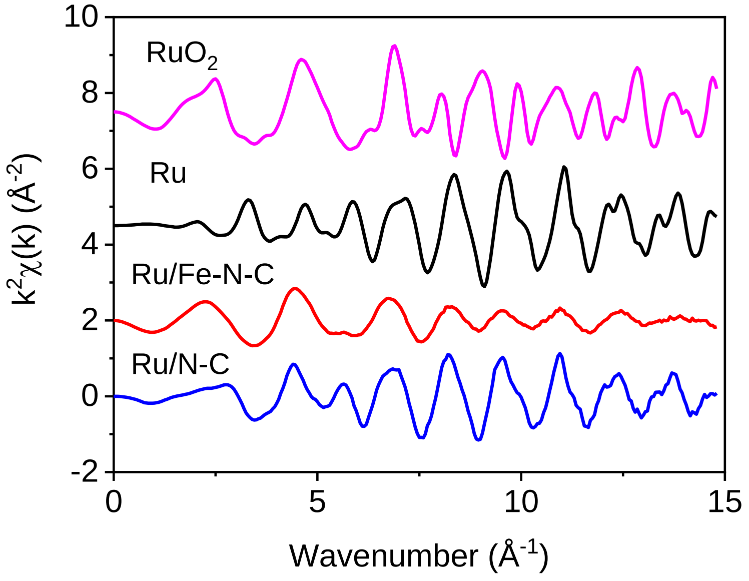

Figure S12 Ru K-edge EXAFS spectra of Ru/Fe-N-C, Ru/N-C, Ru and RuO_2 in k-space.

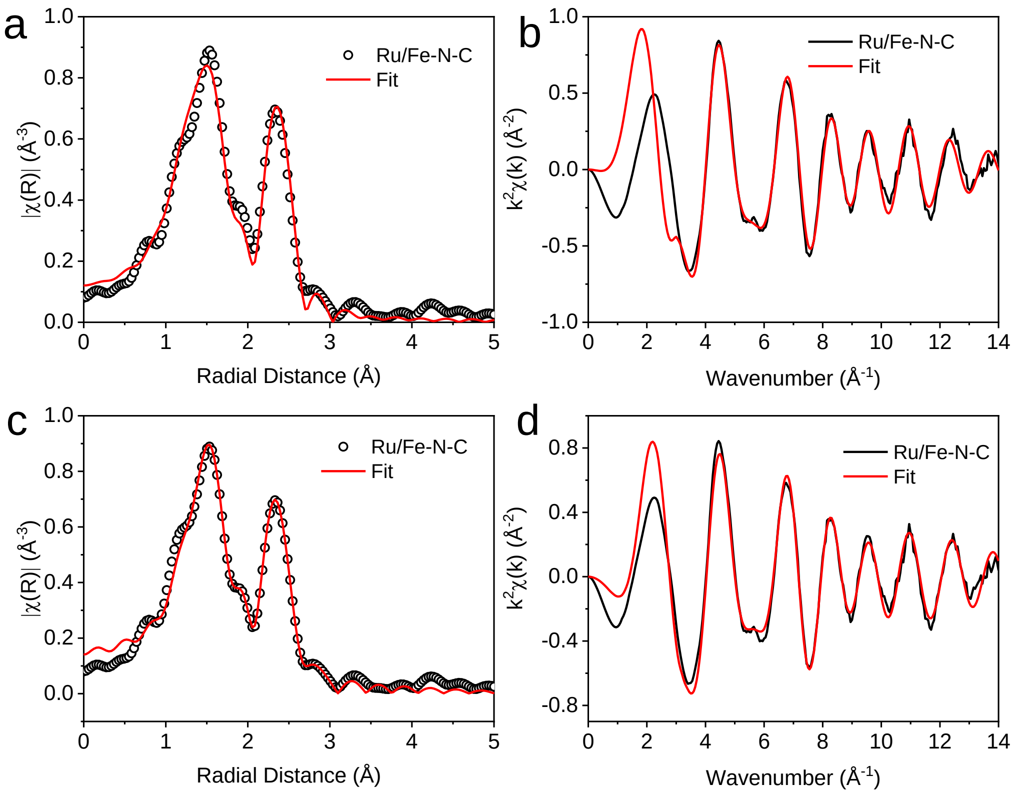

Figure S13 The EXAFS R-space fitting curve (red) and the experimental one (black cycles) of Ru/Fe-N-C (a) without Ru-Fe (c) with Ru-Fe. The EXAFS k-space fitting curve (red) and the experimental one (black) of Ru/Fe-N-C (b) without Ru-Fe (d) with Ru-Fe.

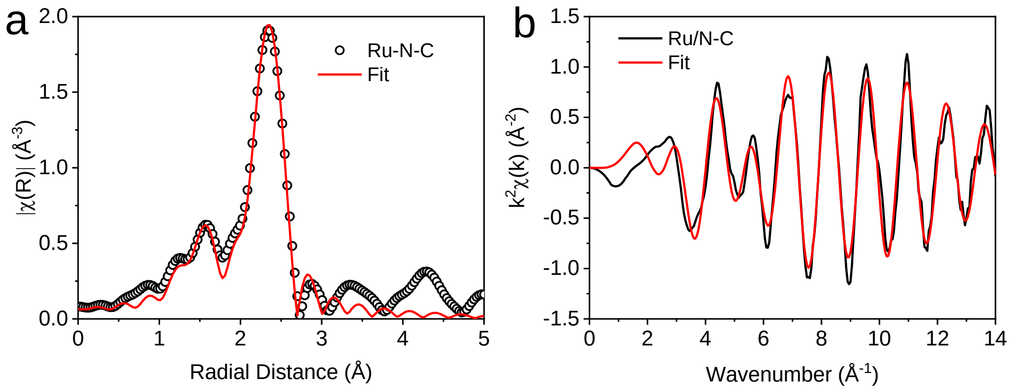

Figure S14 (a) The EXAFS R-space fitting curve (red) and the experimental one (black cycles) of Ru/N-C. (b) The EXAFS k-space fitting curve (red) and the experimental one (black) of Ru/N-C.

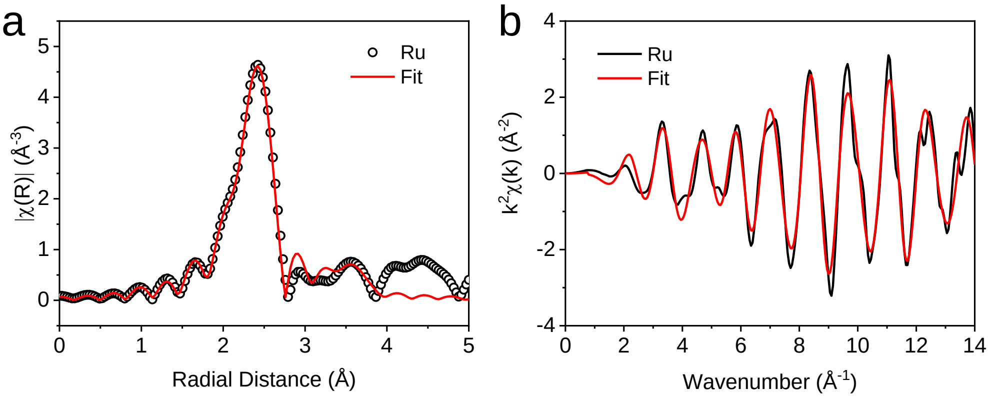

Figure S15 (a) The EXAFS R-space fitting curve (red) and the experimental one (black cycles) of Ru metal. (b) The EXAFS k-space fitting curve (red) and the experimental one (black) of Ru metal.

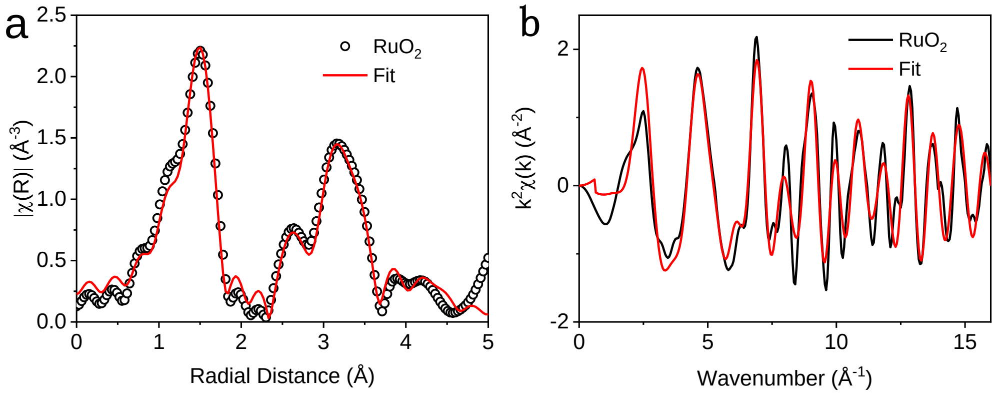

Figure S16 (a) The EXAFS R-space fitting curve (red) and the experimental one (black cycles) of RuO_2. (b) The EXAFS k-space fitting curve (red) and the experimental one (black) of RuO_2.

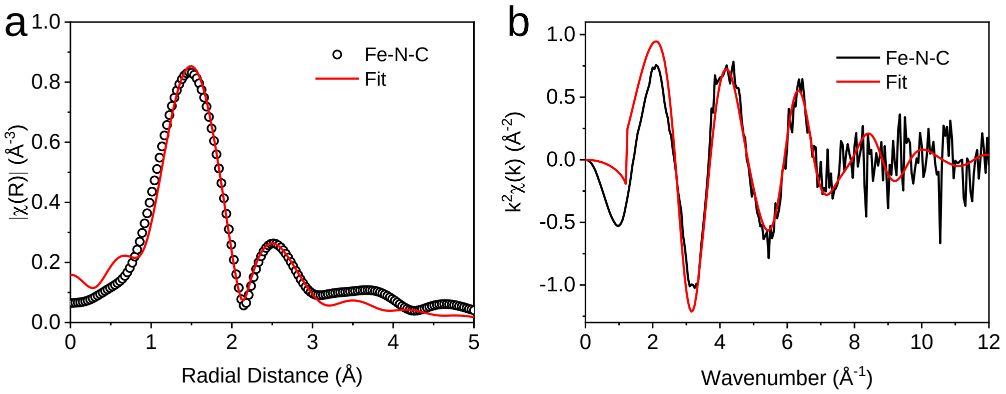

Figure S17 (a) The EXAFS R-space fitting curve (red) and the experimental one (black cycles) of Fe-N-C. (b) The EXAFS k-space fitting curve (red) and the experimental one (black) of Fe-N-C.

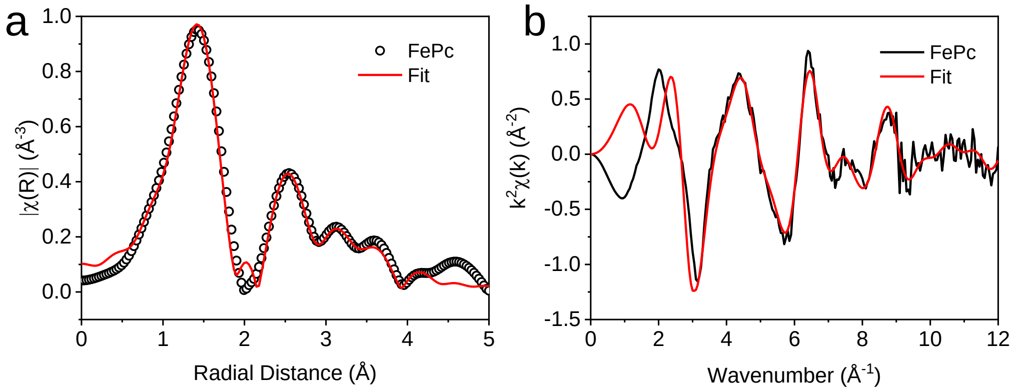

Figure S18 (a) The EXAFS R-space fitting curve (red) and the experimental one (black cycles) of FePc. (b) The EXAFS k-space fitting curve (red) and the experimental one (black) of FePc.

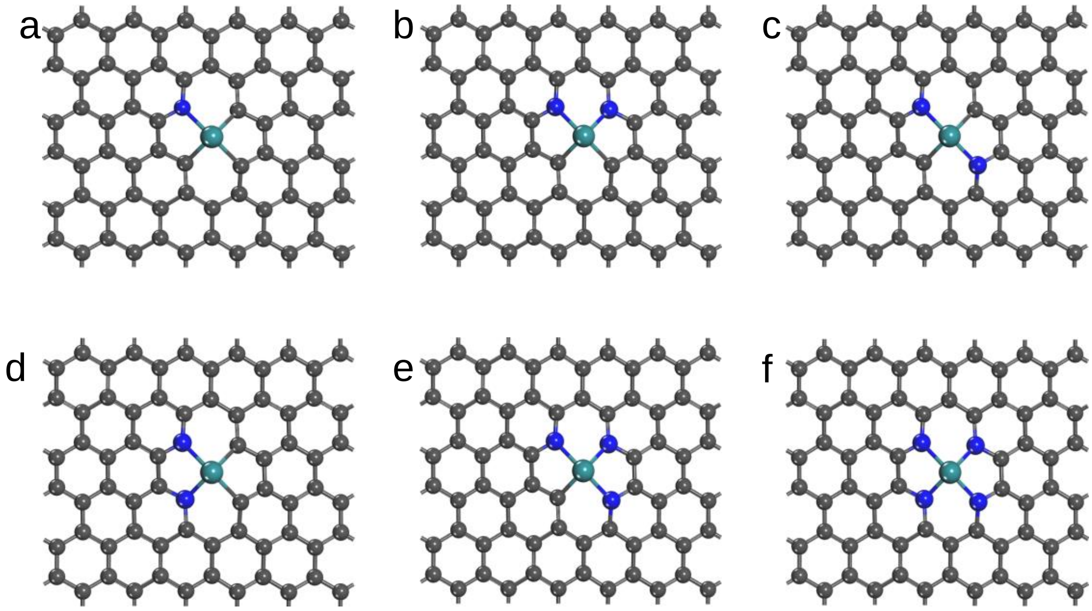

Figure S19 Atomic configurations of simulated RuN_1C_3 (a), RuN_2C_2-1 (b), RuN_2C_2-2 (c), RuN_2C_2-3 (d), RuN_3C_1 (e) and RuN_4 (f).

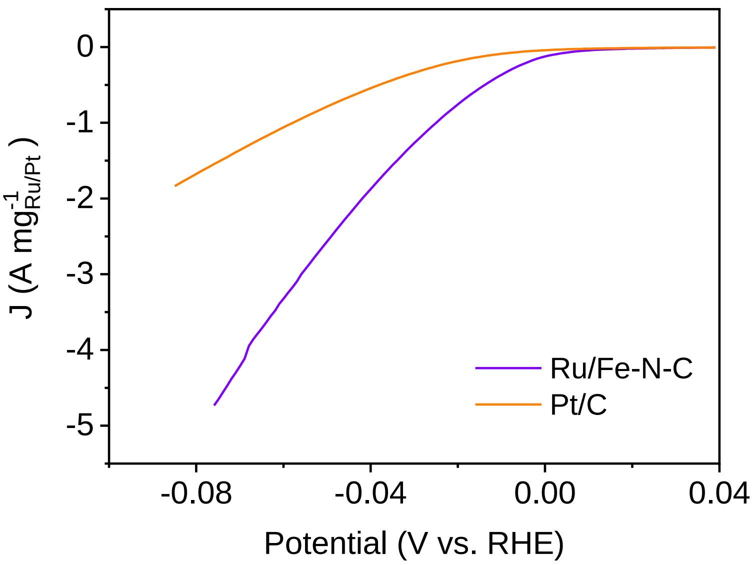

Figure S20 Mass activity of Ru/Fe-N-C and Pt/C.

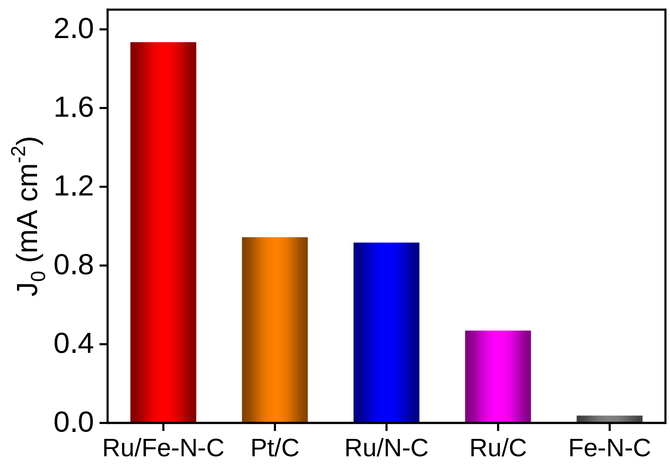

Figure S21 The exchange current density (J_0) of Ru/Fe-N-C, Pt/C, Ru/N-C, Ru/C and Fe-N-C.

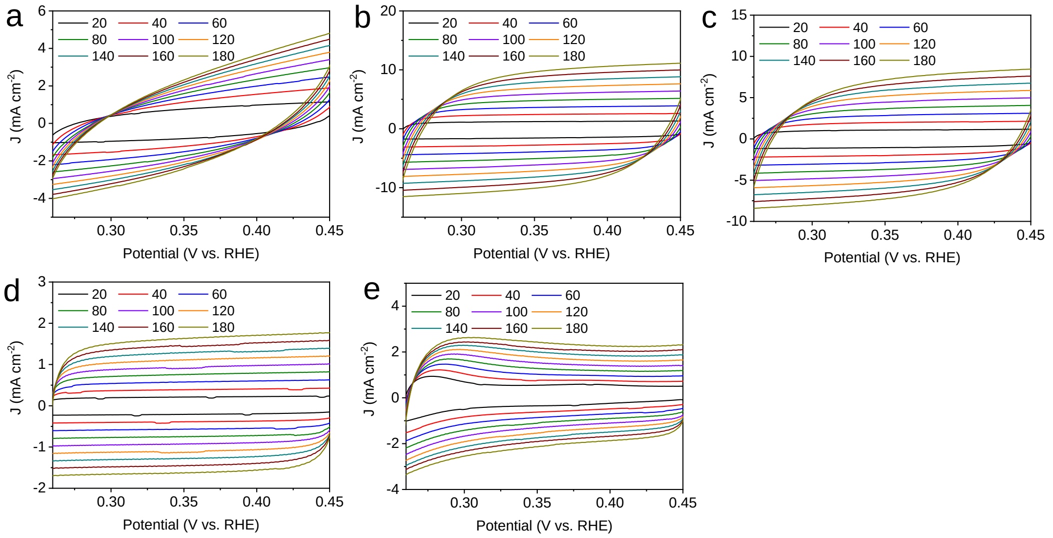

Figure S22 Cyclic Voltammetry curves of Fe-N-C (a), Ru/Fe-N-C (b), Ru/N-C (c), Ru/C (d) and commercial Pt/C (e) catalysts with various scan rates in 1M KOH solution. The capacitive currents were collected at 0.35 V vs. RHE in potential range where no Faradaic processes were present.

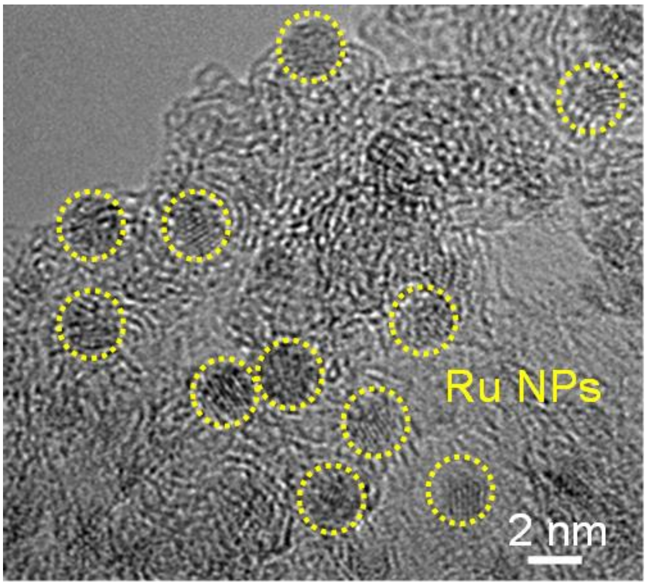

Figure S23 HRTEM image of Ru/Fe-N-C after 5000 CV.

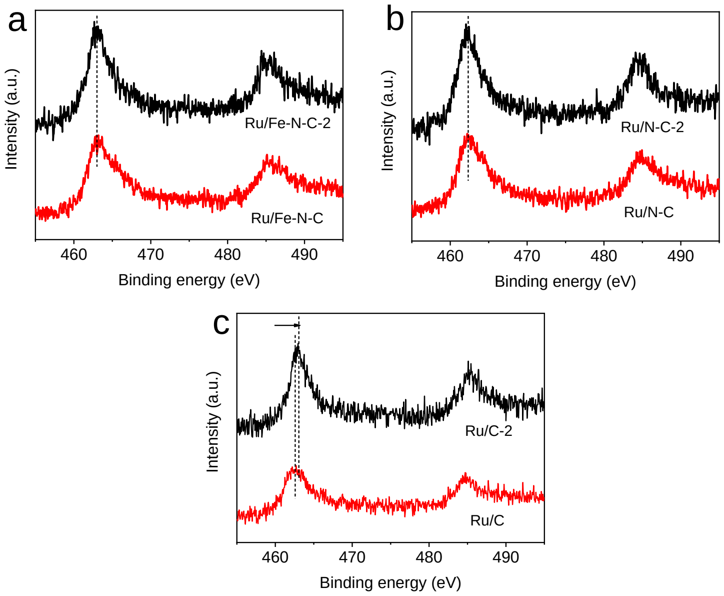

Figure S24. The Ru 3p XPS spectrum of Ru/Fe-N-C (a), Ru/N-C (b) and Ru/C (c) in their initial state and after long-term exposure to air.

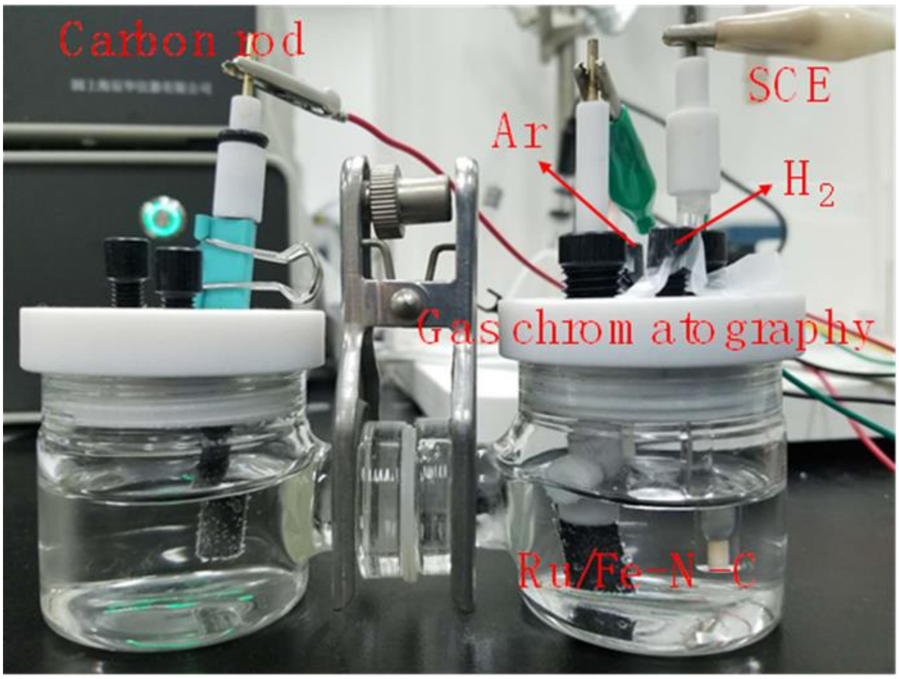

Figure S25 The details of hydrogen evolution Faradaic efficiency measurement in 1M KOH.

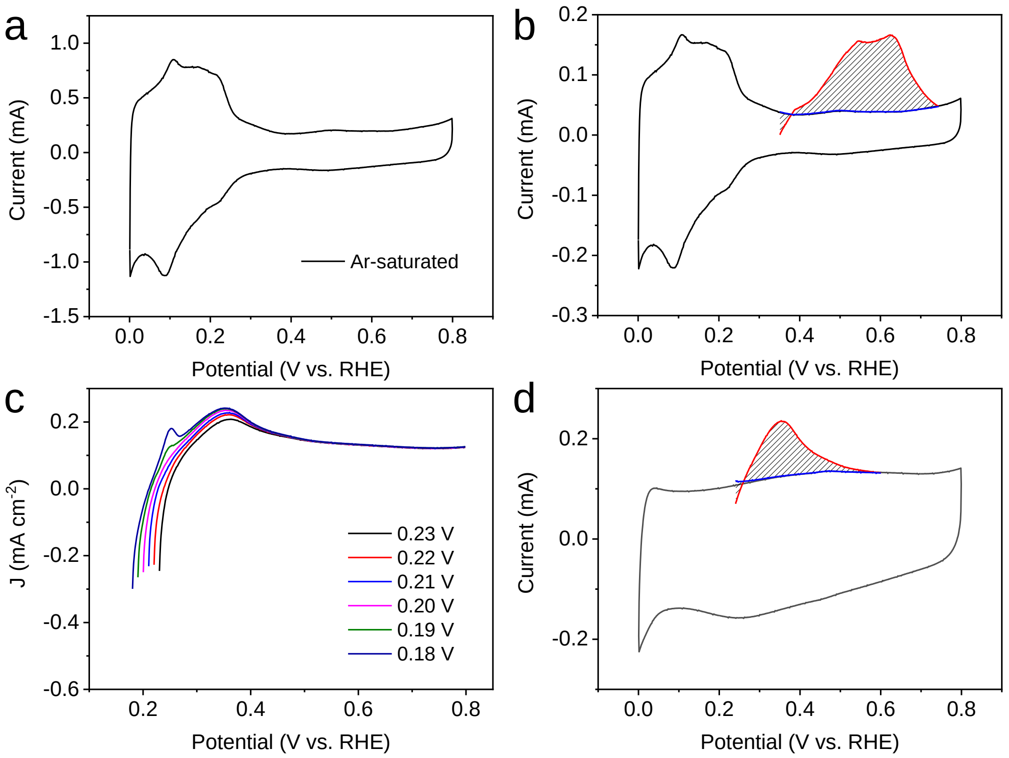

Figure S26 (a) Cycling voltammetry of Pt/C in 0.5 M H_2SO_4. (b) Cu UPD in 0.5 M H_2SO_4 in the absence (blue line) and presence (red line) of 5 mM $CuSO_4$ on Pt/C. The electrode was polarized at 0.205 V for 100 s to form the UPD layer. (c) Cu UPD in 0.5 M H_2SO_4 in the presence of 5 mM $CuSO_4$ on Ru/Fe-N-C. The electrode was polarized at 0.230, 0.220, 0.210, 0.200, 0.190 and 0.180 V for 100 s to form the UPD layers, respectively. (d) Cu UPD in 0.5 M H_2SO_4 in the absence (blue line) and presence (red line) of 5 mM $CuSO_4$ on Ru/Fe-N-C. The electrode was polarized at 0.200 V for 100 s to form the UPD layer. Scan rate: 10 mV s^{-1}.

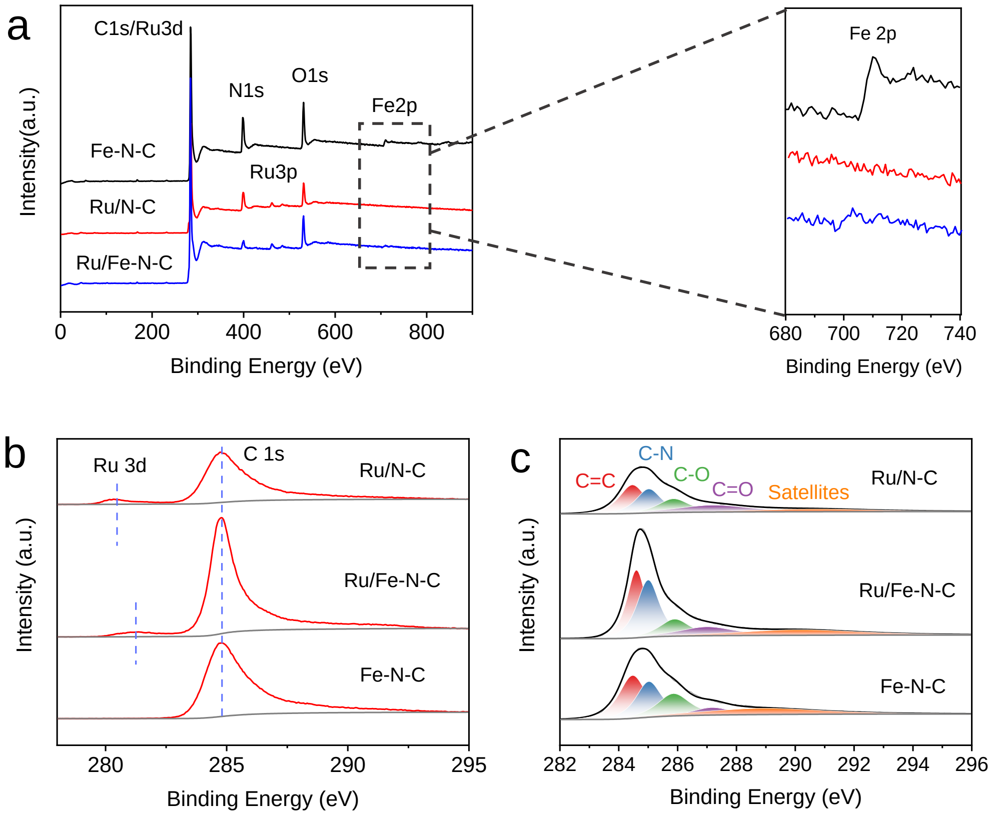

Figure S27 The XPS survey spectra (a), Ru 3d spectra (b) and C 1s spectra (c) of Ru/Fe-N-C, Ru/N-C and Fe-N-C.

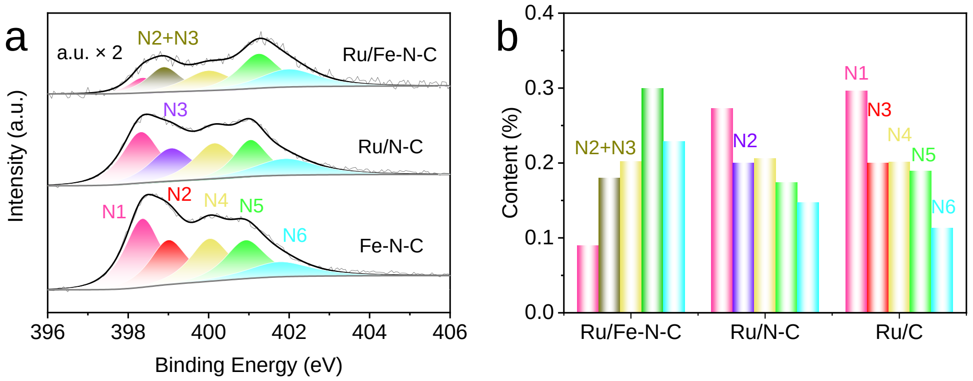

Figure S28 N1s XPS spectra (a) and corresponding N content of Ru/Fe-N-C, Ru/N-C and Fe-N-C (N1, N2, N3, N4, N5 and N6 are corresponding to pyrinidic N, Fe-N, Ru-N, pyrrolic N, graphitic N and N-oxides, respectively.).

N 1s spectrum of Ru/Fe-N-C displays pyridinic−N (398.2 eV), Ru/Fe-N bonding (398.9 eV), pyrrolic−N (400.1 eV), graphitic−N (401.2 eV), and oxidized–N (402.8 eV). Remarkably, the graphitic−N (~401.2 eV) becomes dominant in Ru/Fe-N-C compared to Fe-N-C, favorably improving the electronic conductivity of catalyst, and thus enhancing the HER activity

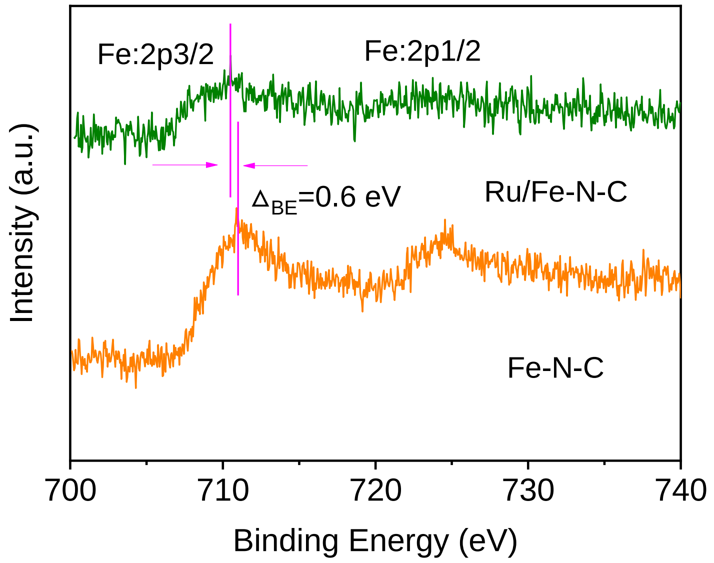

Figure S29 High-resolution Fe 2p XPS spectra of Ru/Fe-N-C and Fe-N-C.

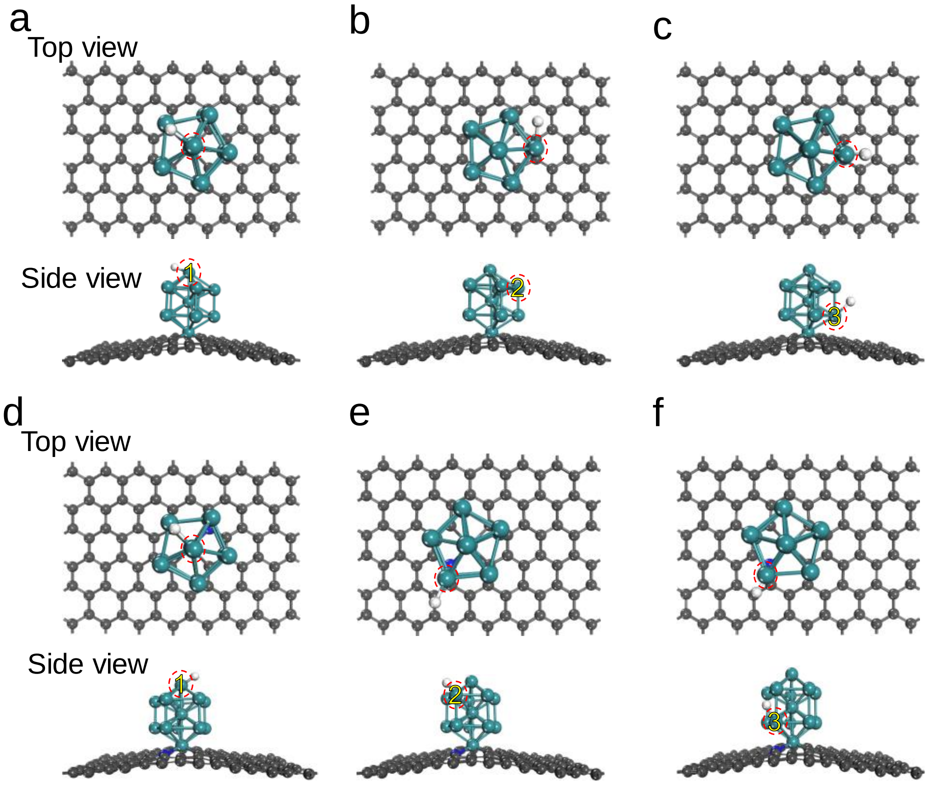

Figure S30 Optimized structure of H adsorbed on Ru1 (a), Ru2 (b) and Ru3 (c) atom of Ru/C system. And optimized structure of H adsorbed on Ru1 (d), Ru2 (e) and Ru3 (f) atom of Ru/N-C system.

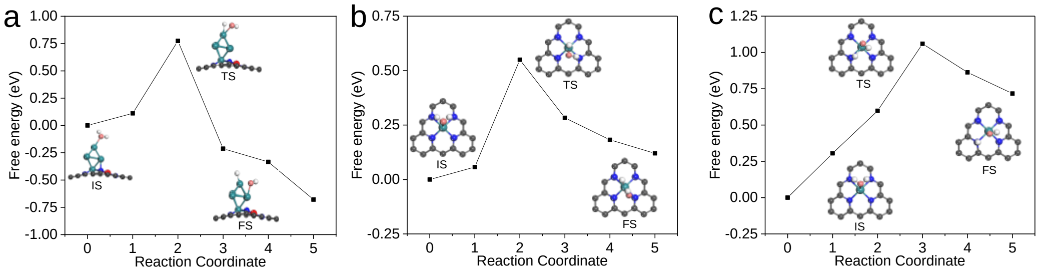

Figure S31 Water dissociation barrier of Ru cluster in Ru/Fe-N-C system (a). And water dissociation barrier for (b) reaction pathway-1 and (c) reaction pathway-2 of Ru-N_4 in Ru/Fe-N-C system. The insets are the structure of the corresponding transition state. The colors of elements are: gray for C, blue for N, red for Fe, pink for O and white for H.

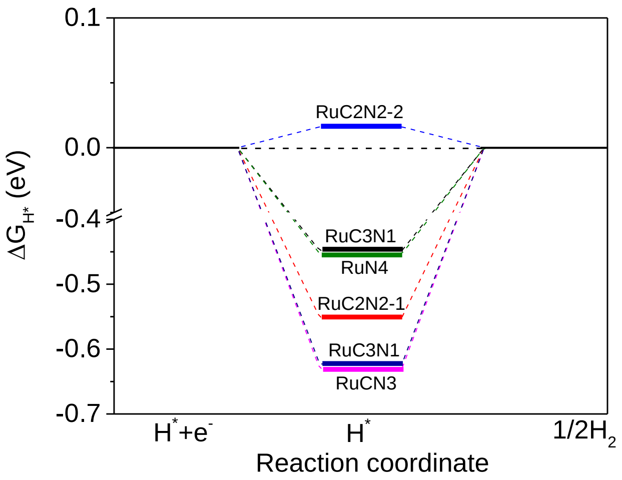

Figure S32 Calculated hydrogen adsorption free energy of multiple active site in Ru single atom system.

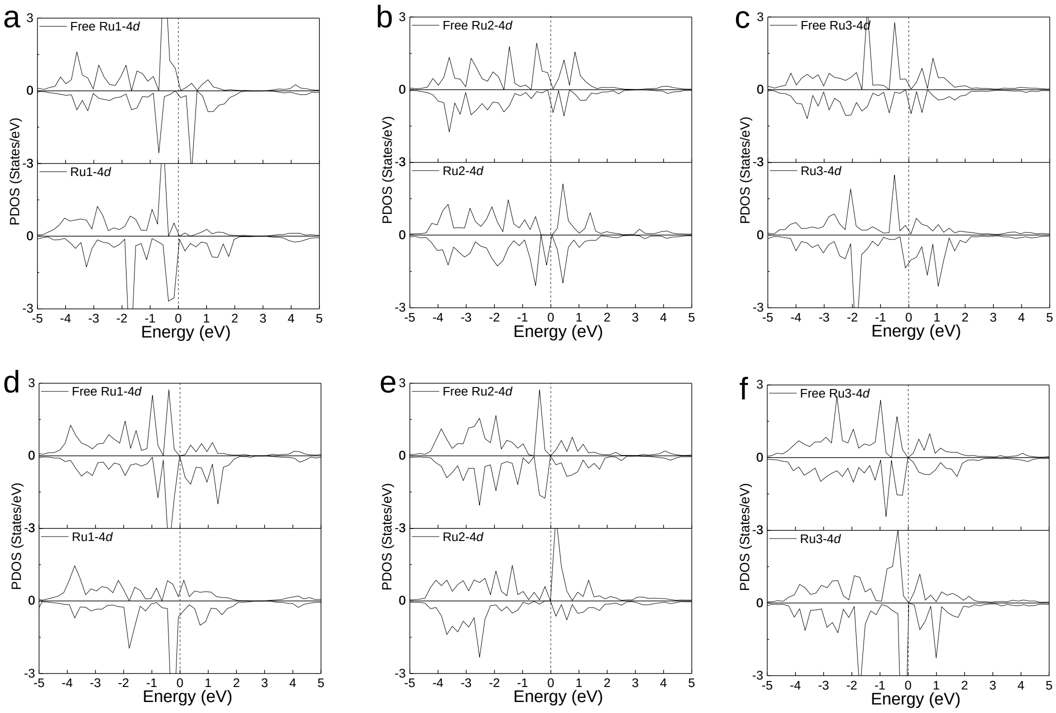

Figure S33 The projected density of state (pDOS) before and after H absorbed on Ru1 (a), Ru2 (b) and Ru3 (c) atom of Ru/C system. And the projected density of state (pDOS) before and after H absorbed on Ru1 (d), Ru2 (e) and Ru3 (f) atom of Ru/N-C system.

Table S1. Element contents for Ru/Fe-N-C determined by above EDS analysis.

Element	Line Type	Apparent Concentration	K Ratio	Wt. %
C	K series	11.77	0.11767	80.9
N	K series	0.12	0.00021	1.6
O	K series	0.31	0.00103	4.2
Fe	K series	0.21	0.00209	1.7
Ru	K series	1.41	0.01409	11.5
Total	K series	-	-	100.00

Table S2 Structural properties of the representative catalysts.

catalyst	BET surface area ($m^2\ g^{-1}$)	Pore size (nm)	Pore volume ($m^3\ g^{-1}$)
Fe-N-C	840	8.7	1.8
Ru/Fe-N-C	810	9.3	1.8
Ru/N-C	880	10	2.1
Ru/ C	894	9.6	2.0

Table S3 Structure parameters (CN: coordination number; R: distance; σ^2: mean-square disorder; $\triangle E_0$: energy shift) of Ru/Fe-N-C, Ru/N-C, RuO_2, bulk Ru metal, Fe-N-C, and standard FePc extracted from the EXAFS fitting. The single digit numbers in parentheses are the last digit errors. The numbers in parentheses for CN are the full errors.

Sample	Path	CN	R(Å)	σ^2(Å^2)	S_0^2	$\triangle E_0$ (eV)	R-factor
Ru/Fe-N-C (without Fe)	Ru-N	6.4 ± 1.1	2.02 ± 0.01	0.0119(4)	1.9(3)	-4.9(5)	0.0227
	Ru-Ru	1.8 ± 0.7	2.66 ± 0.03	0.0046(3)	0.22(3)		
	Ru-Ru	1.8 ± 0.7	2.72 ± 0.03	0.0099(7)	0.22(3)		
	Ru-C	12.7 ± 2.3	3.07 ± 0.04	0.0269(9)	1.9(3)		
Ru/Fe-N-C (with Fe)	Ru-N	3.8 ± 0.3	2.03 ± 0.01	0.0076(9)	1.1(1)	-0.9(1)	0.0070
	Ru-Fe	1± 0	2.60 ± 0.02	0.0200(7)	1.1(1)		
	Ru-Ru	1.8 ± 0.3	2.65 ± 0.01	0.0033(5)	0.2(3)		
	Ru-Ru	1.8 ± 0.3	2.76 ± 0.02	0.0062(8)	0.2(3)		
	Ru-C	1.8 ± 0.3	2.89 ± 0.03	0.0080(4)	1.1(2)		
Ru/N-C	Ru-N	1.2 ± 0.2	2.00 ± 0.03	0.0033(8)	0.37(5)	-6.6(6)	0.0128
	Ru-Ru	3.9 ±	2.64 ±	0.0063(1)	0.45(1)		

		0.01	0.02				
	Ru-Ru	3.9 ± 0.01	2.70 ± 0.02	0.0035(2)	0.45(1)		
Fe-N-C	Fe-N	4	2.03 ± 0.02	0.0096(9)	1.3(1)	5.5(5)	0.0166
	Fe-C	8	3.06 ± 0.04	0.0150(2)			
	Fe-N-C	16	3.28 ± 0.07	0.0136(1)			
FePc	Fe-N	4	1.93 ± 0.01	0.0076(3)	1.2(2)	-3.4(8)	0.0154
	Fe-C	8	2.97 ± 0.02	0.0071(4)			
	Fe-N-C	16	3.14 ± 0.02	0.0029(2)			
	Fe-N	4	3.37 ± 0.02	0.0057(5)			
	Fe-N-N	16	3.86 ± 0.02	0.0008(5)			
	Fe-N-N	4	3.86 ± 0.02	0.0008(5)			
	Fe-C	6	4.20 ± 0.03	0.0155(8)			
	Fe-N-C	12	4.22 ± 0.03	0.0093(1)			
Ru metal	Ru-Ru	6	2.64 ± 0.01	0.0026(9)	0.73(6)	2.4(9)	0.0164
	Ru-Ru	6	2.70 ±	0.0020(7)			

			0.01				
	Ru-Ru	6	3.77 ± 0.01	0.0038(2)			
	Ru-Ru-Ru	36	3.98 ± 0.01	0.0031(1)			
	Ru-O	2	1.95 ± 0.01	0.0010(4)			
	Ru-O	4	1.99 ± 0.01	0.0036(3)			
	Ru-Ru	2	3.12 ± 0.01	0.0024(4)			
	Ru-Ru	8	3.55 ± 0.01	0.0027(6)			
	Ru-O-Ru	16	3.74 ± 0.01	0.0024(5)			
RuO_2 bulk	Ru-O	2	3.90 ± 0.01	0.0005(3)	0.94(9)	1.6(2)	0.0165
	Ru-O	4	3.98 ± 0.01	0.0007(2)			
	Ru-O-O	4	4.43 ± 0.01	0.0008(5)			
	Ru-Ru	4	4.51 ± 0.01	0.0037(2)			
	Ru-O	8	4.63 ± 0.01	0.0082(2)			
	Ru-O-O	16	4.70 ± 0.01	0.0341(1)			

Table S4 The calculated formation energy of RuN_xC_y (x+y≤4) structures in Ru/Fe-N-C.

Structure	Formation energy
RuN_1C_3	5.448
RuN_2C_2-1	4.75
RuN_2C_2-2	4.519
RuN_2C_2-3	5.157
RuN_3C_1	3.985
RuN_4	2.871

Table S5 The calculated average formation energy and charge transfer between Ru nanoclusters and substrate of Ru/C, Ru/N-C and Ru/Fe-N-C structure.

Structure	Average formation energy	Charge
Ru/C	0.251	0.629
Ru/N-C	0.248	0.664
Ru/Fe-N-C	0.224	0.901

Table S6 Summary of some recently reported HER electrocatalysts in 1M KOH electrolyte.

Catalyst	Electrolyte	Overpotential at 10 mA cm^{-2} (mV)	Tafel slope (mV dec^{-1})
Ru/Fe-N-C	1M KOH	9	28
NiFeRu-LDH[2]	1M KOH	29	31
Cu NDs/Ni_3S_2 NTs-CFs[3]	1M KOH	128	76.2
NC/CuCo/$CuCoO_x$[4]	1M KOH	112	55
EG/$Co_{0.85}$Se/NiFeLDH[5]	1M KOH	260	160
Se-(NiCo)S/OH[6]	1M KOH	101	87.3
Ru–MoO_2[7]	1M KOH	29	31
Ni_2P NPs/CC[8]	1M KOH	71	73
Ni@Ni_2P−Ru[9]	1M KOH	31	41
CoP/NCNHP[10]	1M KOH	115	66
Ru_2P@NPC[11]	1M KOH	52	69
NH_2-BP[12]	1M KOH	290	63
NiCu@C[13]	1M KOH	74	94.5
Ni_2P@NPCNFs[14]	1M KOH	104.2	79.7
P-Co_3O_4[15]	1M KOH	120	52
Mo_2N–Mo_2C/HGr[16]	1M KOH	154	68
Cu@NiFe LDH[17]	1M KOH	116	58.9
Co-Ni_3N[18]	1M KOH	194	156
MoB/g-C_3N_4[19]	1M KOH	133	46
A-CoPt-NC[20]	1M KOH	50	48
NP-MoS_2/CC[21]	1M KOH	78	51.6
Co/b-Mo_2C@N-CNTs[22]	1M KOH	170	92

Ru/NG-750[23]	1M KOH	8	30
Ru@C_2N[24]	1M KOH	17	38
RuCoP[25]	1M KOH	23	37
Co-substituted Ru[26]	1M KOH	13	29
[Ru(SA)+Ru(NP)@Ru Nx@GN]/GN[27]	1M KOH	7	20
Ru-NC-700[28]	1M KOH	12	-
Ru@CQDs[29]	1M KOH	10	47
**RuCo@NC[30]**	1M KOH	28	31
**Ru@CN[31]**	1M KOH	32	53
**RuSAs + RuNPs@MHC[32]**	1M KOH	7	29
**Cu/Ru@G_N[33]**	1M KOH	8	20
**RuNi/CQDs[34]**	1M KOH	13	40
**BPed-Pt/GR[35]**	1M KOH	21	46.9
**Ru@SC-CDs[36]**	1M KOH	29	57

Table S7 TOF values of Ru-based electrocatalysts in 1M KOH solution.

Catalyst	Electrolyte	TOF (H_2 s^{-1})
Ru/Fe-N-C (This work)	1M KOH	3.6 at η=25 mV; 8.9 at η=50 mV
Ru@C_2N[24]	1M KOH	0.76 at η=25 mV; 1.66 at η=50 mV
Ru/NC[37]	1M KOH	4.55 at η=100 mV
Ru/NG-750[23]	1M KOH	0.35 at η=100 mV
RuCoP[25]	1M KOH	7.26 at η=100 mV
Co-substituted Ru[26]	1M KOH	2.15 at η=30 mV; 6.39 at η=60 mV
Cu/Ru@G_N[33]	1M KOH	1.47 at η=20 mV
RuNi/CQDs[34]	1M KOH	5.03 at η=100 mV
Ru@SC-CDs[36]	1M KOH	0.56 at η=100 mV

Table **S8** Elemental composition of the representative catalysts from XPS.

Catalyst	Ru (wt. %)	Fe (wt. %)	N (wt. %)	C (wt. %)	O (wt. %)
Fe-N-C	0	3.7	14.5	70.4	11.4
Ru0.05/Fe-N-C	0.4	2.1	5.9	82.8	8.8
Ru0.1/Fe-N-C	0.89	1.96	4.7	79.95	12.5
Ru/Fe-N-C	1.42	1.98	3.67	84.03	8.9
Ru/N-C	2.88	0	12.7	74.51	9.91
Ru/C	2.62	0	0	81.77	15.61
Ru0.3/Fe-N-C	2.29	2.15	4.34	79.52	11.7

Table S9 Ru content of the catalysts from ICP-AES experiment.

Catalyst	Ru (wt. %)
Ru/Fe-N-C	4.92
Ru/N-C	8.06
Ru/C	8.47

References for supporting information:

[1] J. Pampel, T.-P. Fellinger, *Adv. Energy Mater.* **2016**, *6*, 1502389.
[2] G. Chen, T. Wang, J. Zhang, P. Liu, H. Sun, X. Zhuang, M. Chen, X. Feng, *Adv. Mater.* **2018**, *30*, 1706279.
[3] J. X. Feng, J. Q. Wu, Y. X. Tong, G. R. Li, *J. Am. Chem. Soc.* **2018**, *140*, 610.
[4] J. Hou, Y. Sun, Y. Wu, S. Cao, L. Sun, *Adv. Funct. Mater.* **2018**, *28*, 1704447.
[5] Y. Hou, M. R. Lohe, J. Zhang, S. Liu, X. Zhuang, X. Feng, *Energ. Environ. Sci.* **2016**, *9*, 478.
[6] C. Hu, L. Zhang, Z. J. Zhao, A. Li, X. Chang, J. Gong, *Adv. Mater.* **2018**, *30*, e1705538.
[7] P. Jiang, Y. Yang, R. Shi, G. Xia, J. Chen, J. Su, Q. Chen, *J. Mater. Chem. A* **2017**, *5*, 5475.
[8] Y. Lin, L. He, T. Chen, D. Zhou, L. Wu, X. Hou, C. Zheng, *J. Mater. Chem. A* **2018**, *6*, 4088.
[9] Y. Liu, S. Liu, Y. Wang, Q. Zhang, L. Gu, S. Zhao, D. Xu, Y. Li, J. Bao, Z. Dai, *J. Am. Chem. Soc.* **2018**, *140*, 2731.
[10] Y. Pan, K. Sun, S. Liu, X. Cao, K. Wu, W. C. Cheong, Z. Chen, Y. Wang, Y. Li, Y. Liu, D. Wang, Q. Peng, C. Chen, Y. Li, *J. Am. Chem. Soc.* **2018**, *140*, 2610.
[11] Z. Pu, I. S. Amiinu, Z. Kou, W. Li, S. Mu, *Angew. Chem. Int. Ed.* **2017**, *56*, 11559.
[12] L. Shao, H. Sun, L. Miao, X. Chen, M. Han, J. Sun, S. Liu, L. Li, F. Cheng, J. Chen, *J. Mater. Chem. A* **2018**, *6*, 2494.
[13] Y. Shen, Y. Zhou, D. Wang, X. Wu, J. Li, J. Xi, *Adv. Energy Mater.* **2018**, *8*, 1701759.
[14] M. Q. Wang, C. Ye, H. Liu, M. Xu, S. J. Bao, *Angew. Chem. Int. Ed.* **2018**, *57*, 1963.
[15] Z. Xiao, Y. Wang, Y.-C. Huang, Z. Wei, C.-L. Dong, J. Ma, S. Shen, Y. Li, S. Wang, *Energ. Environ. Sci.* **2017**, *10*, 2563.
[16] H. Yan, Y. Xie, Y. Jiao, A. Wu, C. Tian, X. Zhang, L. Wang, H. Fu, *Adv. Mater.* **2018**, *30*, 1704156.
[17] L. Yu, H. Zhou, J. Sun, F. Qin, F. Yu, J. Bao, Y. Yu, S. Chen, Z. Ren, *Energ. Environ. Sci.* **2017**, *10*, 1820.

[18]C. Zhu, A. L. Wang, W. Xiao, D. Chao, X. Zhang, N. H. Tiep, S. Chen, J. Kang, X. Wang, J. Ding, J. Wang, H. Zhang, H. J. Fan, *Adv. Mater.* **2018**, *30*, e1705516.
[19]Z. Zhuang, Y. Li, Z. Li, F. Lv, Z. Lang, K. Zhao, L. Zhou, L. Moskaleva, S. Guo, L. Mai, *Angew. Chem. Int. Ed.* **2018**, *57*, 496.
[20]L. Zhang, Y. Jia, H. Liu, L. Zhuang, X. Yan, C. Lang, X. Wang, D. Yang, K. Huang, S. Feng, X. Yao, *Angew. Chem. Int. Ed.* **2019**, *58*, 9404.
[21]K. Sun, L. Zeng, S. Liu, L. Zhao, H. Zhu, J. Zhao, Z. Liu, D. Cao, Y. Hou, Y. Liu, Y. Pan, C. Liu, *Nano Energy* **2019**, *58*, 862.
[22]T. Ouyang, Y. Q. Ye, C. Y. Wu, K. Xiao, Z. Q. Liu, *Angew. Chem. Int. Ed.* **2019**, *58*, 4923.
[23]R. Ye, Y. Liu, Z. Peng, T. Wang, A. S. Jalilov, B. I. Yakobson, S. H. Wei, J. M. Tour, *ACS Appl. Mater. Inter.* **2017**, *9*, 3785.
[24]J. Mahmood, F. Li, S. M. Jung, M. S. Okyay, I. Ahmad, S. J. Kim, N. Park, H. Y. Jeong, J. B. Baek, *Nat. Nanotechnol.* **2017**, *12*, 441.
[25]J. Xu, T. Liu, J. Li, B. Li, Y. Liu, B. Zhang, D. Xiong, I. Amorim, W. Li, L. Liu, *Energ. Environ. Sci.* **2018**, *11*, 1819.
[26]J. Mao, C. T. He, J. Pei, W. Chen, D. He, Y. He, Z. Zhuang, C. Chen, Q. Peng, D. Wang, Y. Li, *Nat. Commun.* **2018**, *9*, 4958.
[27]J. N. Tiwari, A. M. Harzandi, M. Ha, S. Sultan, C. W. Myung, H. J. Park, D. Y. Kim, P. Thangavel, A. N. Singh, P. Sharma, S. S. Chandrasekaran, F. Salehnia, J. W. Jang, H. S. Shin, Z. Lee, K. S. Kim, *Adv. Energy Mater.* **2019**, *9*, 1900931
[28]B. Lu, L. Guo, F. Wu, Y. Peng, J. E. Lu, T. J. Smart, N. Wang, Y. Z. Finfrock, D. Morris, P. Zhang, N. Li, P. Gao, Y. Ping, S. Chen, *Nat. Commun.* **2019**, *10*, 631.
[29]W. Li, Y. Liu, M. Wu, X. Feng, S. A. T. Redfern, Y. Shang, X. Yong, T. Feng, K. Wu, Z. Liu, B. Li, Z. Chen, J. S. Tse, S. Lu, B. Yang, *Adv. Mater.* **2018**, *30*, e1800676.
[30]J. Su, Y. Yang, G. Xia, J. Chen, P. Jiang, Q. Chen, *Nat. Commun.* **2017**, *8*, 14969.
[31]J. Wang, Z. Wei, S. Mao, H. Li, Y. Wang, *Energ. Environ. Sci.* **2018**, *11*, 800.
[32]J. N. Tiwari, N. K. Dang, S. Sultan, P. Thangavel, H. Y. Jeong, K. S. Kim, Nat. Sustain. 2020, 3, 556.
[33] A. M. Harzandi, S. Shadman, M. Ha, C. W. Myung, D. Y. Kim, H. J. Park, S. Sultan, W.-S. Noh, W. Lee, P. Thangavel, W. J. Byun, S.-h. Lee, J. N. Tiwari, T. J. Shin, J.-H. Park, Z. Lee, J. S. Lee, K. S. Kim, Appl. Catal. B 2020, 270, 118896.
[34] Y. Liu, X. Li, Q. Zhang, W. Li, Y. Xie, H. Liu, L. Shang, Z. Liu, Z. Chen, L. Gu, Z. Tang, T. Zhang, S. Lu, Angew. Chem. Int. Ed. 2020, 59, 1718.
[35] X. Wang, L. Bai, J. Lu, X. Zhang, D. Liu, H. Yang, J. Wang, P. K. Chu, S. Ramakrishna, X. F. Yu, Angew. Chem. Int. Ed. 2019, 58, 19060.
[36] Y. Liu, Y. Yang, Z. Peng, Z. Liu, Z. Chen, L. Shang, S. Lu, T. Zhang, Nano Energy 2019, 65, 104023.
[37]J. Zhang, P. Liu, G. Wang, P. P. Zhang, X. D. Zhuang, M. W. Chen, I. M. Weidinger, X. L. Feng, *J. Mater. Chem. A* **2017**, *5*, 25314.
